# Protective immunity differs between routes of administration of attenuated malaria parasites independent of parasite liver load

**DOI:** 10.1038/s41598-017-10480-1

**Published:** 2017-09-04

**Authors:** Simone Haeberlein, Séverine Chevalley-Maurel, Arifa Ozir-Fazalalikhan, Hester Koppejan, Beatrice M. F. Winkel, Jai Ramesar, Shahid M. Khan, Robert W. Sauerwein, Meta Roestenberg, Chris J. Janse, Hermelijn H. Smits, Blandine Franke-Fayard

**Affiliations:** 1 Department of Parasitology, Leiden University Medical Center, Albinusdreef 2, 2333 ZA Leiden, The Netherlands; 2 Department of Infectious Diseases, Leiden University Medical Center, Albinusdreef 2, 2333 ZA Leiden, The Netherlands; 3 Department of Medical Microbiology, Radboud University Medical Center, Geert-Grooteplein 28, 6525 GA Nijmegen, The Netherlands; 4 Present Address: Institute of Parasitology, Justus-Liebig-University Giessen, Schubertstrasse 81, 35392 Giessen, Germany

## Abstract

In humans and murine models of malaria, intradermal immunization (ID-I) with genetically attenuated sporozoites that arrest in liver induces lower protective immunity than intravenous immunization (IV-I). It is unclear whether this difference is caused by fewer sporozoites migrating into the liver or by suboptimal hepatic and injection site-dependent immune responses. We therefore developed a *Plasmodium yoelii* immunization/boost/challenge model to examine parasite liver loads as well as hepatic and lymph node immune responses in protected and unprotected ID-I and IV-I animals. Despite introducing the same numbers of genetically attenuated parasites in the liver, ID-I resulted in lower sterile protection (53–68%) than IV-I (93–95%). Unprotected mice developed less sporozoite-specific CD8^+^ and CD4^+^ effector T-cell responses than protected mice. After immunization, ID-I mice showed more interleukin-10-producing B and T cells in livers and skin-draining lymph nodes, but fewer hepatic CD8 memory T cells and CD8^+^ dendritic cells compared to IV-I mice. Our results indicate that the lower protection efficacy obtained by intradermal sporozoite administration is not linked to low hepatic parasite numbers as presumed before, but correlates with a shift towards regulatory immune responses. Overcoming these immune suppressive responses is important not only for live-attenuated malaria vaccines but also for other live vaccines administered in the skin.

## Introduction

Malaria remains a major threat to the lives of more than 3 billion people world-wide. There is a pressing and yet unmet need for an effective vaccine that provides a high degree of sustained protection. Despite decades of clinical testing of (recombinant) sub-unit vaccines, only modest protection has been achieved so far. As a consequence, the interest in whole organism malaria vaccine approaches has been renewed^1–4^.

Induction of complete protective immunity in humans has only been achieved by immunization with live attenuated *Plasmodium* sporozoites^1, 5, 6^ or by (non-attenuated) sporozoites that are administered under chemoprophylaxis^7, 8^. Attenuated sporozoites induce strong protective immune responses both in rodents^9, 10^ and in humans^5, 6, 11^. Injected sporozoites need to be alive and to retain capacity to invade hepatocytes to induce protective immunity. Most immunization studies in rodent models have been conducted using the intravenous (IV) route of administration of sporozoites and only a few studies have analyzed alternative techniques such as intradermal (ID), intramuscular (IM) or subcutaneous (SC) injection of sporozoites^12–18^. However, the latter techniques will be more amenable for large-scale administration to infants in endemic countries. For vaccines in general there is renewed interest in the intradermal route of administration driven by the fact that the dermis and epidermis of human skin are rich in antigen-presenting cells, suggesting that delivery of vaccines to these layers should be more efficient and induce protective immune responses with smaller amounts of vaccine antigen^19^.

Unfortunately, immunization by ID, IM or SC injections of attenuated sporozoites of both rodent (*P. berghei, P. yoelii)* and human (*P. falciparum)* malaria parasites induced lower levels of protective immunity compared to IV administration^16, 20–23^. In rodent malaria models, reduced potency was linked to a lower number of parasites in the liver (30–50 fold) after ID immunization (ID-I) compared to IV immunization (IV-I)^12, 13, 17, 24^. The importance of the number of sporozoites in the liver, i.e. the parasite liver load, for protective immunity is emphasized by the observations that high level protection can be achieved after ID-I provided that sufficiently high numbers of sporozoites are injected^17, 24^. This suggests that induction of protection mainly associates with the number of attenuated sporozoites reaching the liver and infecting hepatocytes^25–31^.

Protective immunity induced by immunization with sporozoites is associated with expansion of IFN-γ producing CD8 memory T cells in the liver^13, 32–35^. Lower CD8 T cell responses were found after ID-I compared to IV-I which was explained by the lower parasite loads in the liver after ID-I^13^. Therefore, it has been speculated that the differences between ID-I and IV-I are the result of fewer parasites entering the liver after ID-I^14^. However, it is unknown whether the differences in protective immunity between ID-I and IV-I can be exclusively explained by differences in parasite liver loads or whether other immunological factors associated with the route of administration of sporozoites can also influence the induction of protective immune responses. Some authors favor the view that sporozoites deposited in the skin use the lymphatic system and thereby pass through lymph nodes to reach the liver^36, 37^.

In order to study the effect of the route of sporozoite administration on development of protective immune responses we developed a mouse model to compare sporozoite IV-I and ID*-*I, parasite liver load and immune responses in protected and in unprotected animals.

Unexpectedly, we found that at similar parasite liver loads, ID immunization resulted in lower protection compared to IV immunization. ID-I mice had less liver CD8 T cell effector responses and a stronger activation of immune cells with regulatory phenotype, compared to IV-I mice. We provide evidence that suboptimal protective efficacy by ID-I compared to IV-I cannot be solely explained by differences in parasite liver load. Our data indicate that the lower protective immunity results from different immune responses induced by ID immunization in skin-draining lymph node and liver compared to IV immunization. This may be the result of an immune-evasion strategy developed by the malaria parasite and understanding and countering this possible immune-evasion strategy should help in development of skin-based vaccination strategies for whole organism approaches of mosquito-borne diseases. Therefore while the skin remains an attractive target for immunization as it possesses an abundance of antigen presenting cells, our study indicates that this route can also negatively affect protective immune responses by generating immunosuppressive or modulatory responses after immunization, which has clear implications beyond malaria vaccines.

## Results

### Reduced protection by ID immunization compared to IV immunization despite similar parasite liver loads

**Figure 1:**
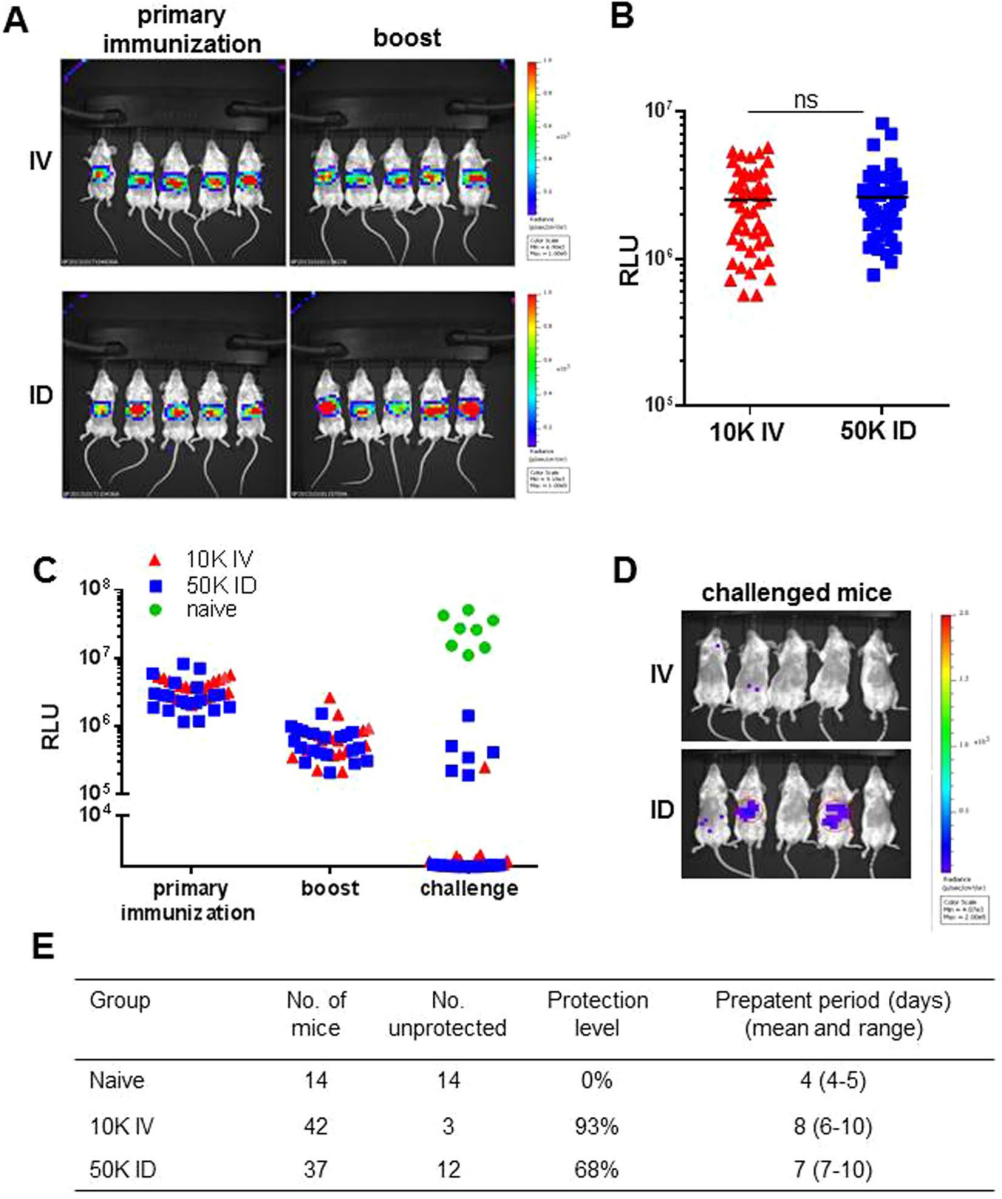
ID immunization with 50 K sporozoites results in same parasite liver load as 10 K IV but determines different outcome of protection. BALB/c mice were immunized with a liver-attenuated luciferase-expressing *P. yoelii* mutant (ΔPyFabBF-GFP-Luc_con_) by intravenous (IV) or intradermal (ID) inoculation of 10 K or 50 K sporozoites, respectively, followed by an IV or ID boost 2 weeks later. 44 h after primary immunization and boost, parasite liver loads were analyzed by *in vivo* imaging of luciferase activity. (**A**) *In vivo* images which show 5 representative mice for each group after primary immunization and boost (parasite load measured as relative luminescence units, RLU, red color indicates high parasite load). (**B**) Summary of luciferase activity of 48 (10 K IV) and 43 (50 K ID) mice after primary immunization from 6 experiments. ns: no significance as determined by unpaired t-test. ns, not significant. (**C**–**E**) BALB/c mice were IV or ID immunized and challenged IV with 10 K luciferase-expressing wild-type (wt) *P. yoelii* sporozoites (Py-GFP-Luc_con_) 2 weeks after boost (see treatment scheme in Fig. S2). 44 h after immunization, boost or challenge, parasite liver loads were analyzed by *in vivo* imaging of luciferase activity. Blood smears were analyzed from day 4–14 after challenge to assess prepatency times. (**C**) Cumulative results from two separate experiments (n = 18, IV inoculation; n = 20, ID inoculation and n = 8, naïve) of liver load measured by luciferase activity. (**D**) *In vivo* images of 5 representative mice per group. (**E**) Protection rate and days of prepatency summarized for 4 experiments. All naïve mice infected with wt parasites developed blood infections.

To investigate whether the low protective immunity after ID-I compared to IV-I can only be explained by differences in liver load we developed a *P. yoelii* mouse model to examine parasite liver loads and immune responses in the same animal. We generated a *P. yoelii* attenuated parasite line that lacks the *fabb/f* gene (PY17X_1126500) and in addition expresses the reporter protein GFP-Luciferase under control of the constitutive *eef1a* promoter (ΔPyFabBF-GFP-Luc_con_ parasites; Fig. S1). This transgenic line shows wild-type (wt) progression through the complete parasite life-cycle^38–40^. Deletion of the *fabb/f* gene in *P. yoelii* results in attenuated parasites that arrest late during liver stage development^41^ and allows for quantification of liver loads by measuring luciferase signals without the need of sacrificing the animal^42^. Using quantification of liver loads by *in vivo* imaging 44 hour after ID and IV administration of attenuated sporozoites we established that administration of 5 times more sporozoites ID (50 K) than IV (10 K), resulted in comparable liver loads and a high percentage of protected mice (>90%) after IV-I, using a primary and one boost immunization. With this regime similar parasite liver loads were obtained both after the primary immunization and the boost immunization 14 days later (Fig. 1).

To compare the level of protection, IV-I and ID-I mice with similar liver loads were challenged 14 days later with 10 K wt-Py-GFP-Luc_con._ sporozoites IV or by bite of mosquito with wt-Py-GFP-Luc_con_. Wt-Py-GFP-Luc_con_ parasites express GFP-Luciferase under control of the constitutive *eef1a* promoter, allowing quantification of parasite liver load after challenge by *in vivo* imaging. Protection was assessed by monitoring blood stage parasitemia in blood smears up to day 14 after challenge (see schedule in Fig. S2). Despite comparable parasite liver load after IV-I and ID-I (Fig. 1C), only 68% of ID-I mice were protected compared to 93% upon IV-I. The ID-I and IV-I unprotected mice showed a comparable prepatent period of blood infection (on average 7 and 8 days, in the ID-I and IV-I mice, respectively (Fig. 1E). In comparison, naïve mice challenged with 10 K sporozoites show a prepatent period of 4 days. A similar difference in protection between IV-I and ID-I mice was obtained when mice were challenged by infectious mosquito bite instead of needle inoculation (Fig. S3).

#### Artesunate treatment of challenged mice to determine immunization-induced immune responses in the liver independent of blood stage infections in unprotected mice

**Figure 2:**
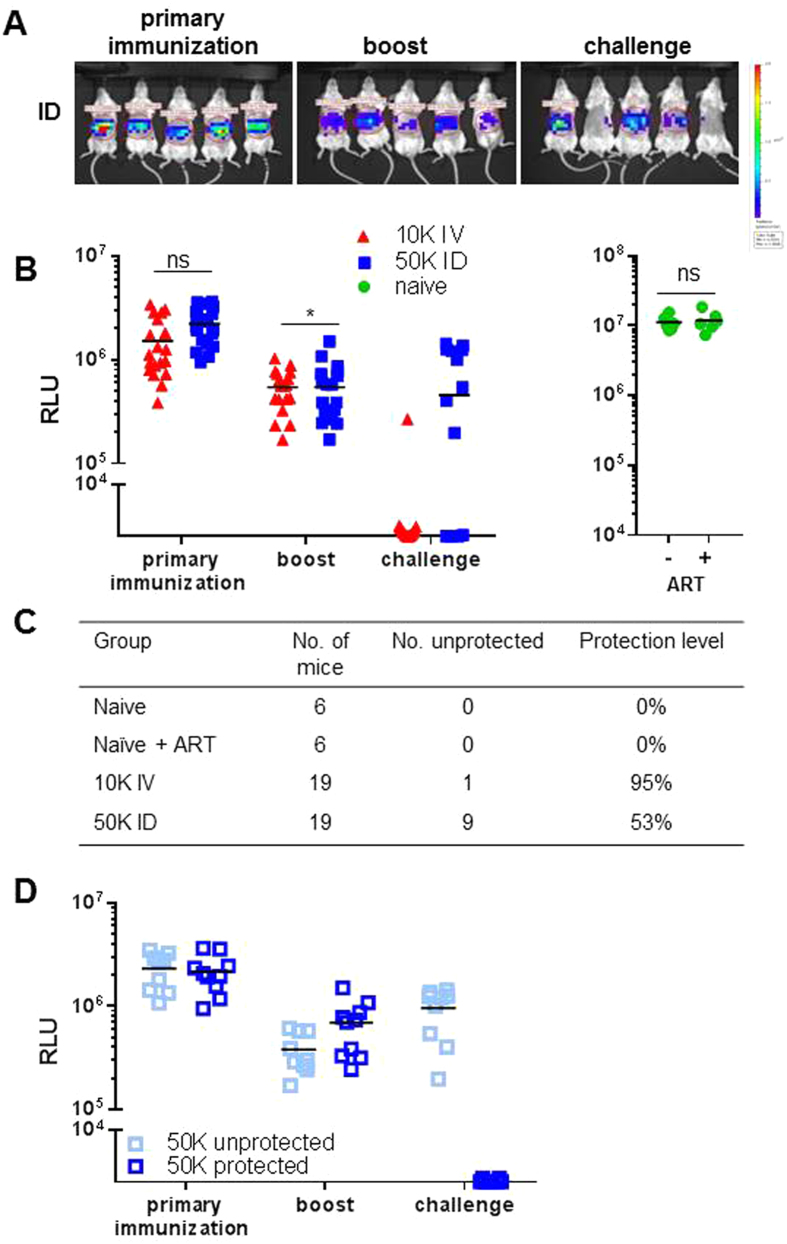
Level of protection in IV and ID immunized mice treated with artesunate (ART) after challenge with wild type parasites. BALB/c mice were immunized by IV or ID route. From the day of wt sporozoite challenge, mice were treated with artesunate to prevent development of blood stages in mice (see treatment scheme in Fig. S4). 44 h after primary immunization, boost or challenge, parasite liver loads were quantified by *in vivo* imaging of luciferase activity. **(A)** Luciferase activity of 5 representative mice from the ID immunized group visualized after primary immunization, boost and challenge. **(B)** Summary of luciferase activity during the immunization/challenge protocol of mice immunized by the IV and ID route, and of challenged naïve mice. **(C)** Protection rates from 2 experiments. Based on the luciferase activity after challenge, mice were grouped into protected (luciferase negative) and unprotected (luciferase positive). ns: no significance, *p < 0.05 as determined by unpaired t-test. **(D)** Mice were immunized by ID route and challenged with wt sporozoites under artesunate treatment. Development of liver stages was analyzed by *in vivo* imaging at 44 h after primary immunization, boost, or challenge. Based on the luciferase activity after challenge, mice were grouped into protected and unprotected. Summary of 19 ID immunized mice.

To assess immunization-induced effects on liver immune responses without interference of immune responses induced by blood stage infections^43, 44^, blood stage development should be avoided after challenge in unprotected mice. To this end, all IV- and ID-I mice were treated with the blood-stage killing drug artesunate starting at the day of challenge with wt sporozoites (see treatment scheme in Fig. S4). Absence of blood stages was confirmed by blood smears up to day 7 after challenge prior to organ collection. In these experiments protection was defined by the level of the parasite liver load in challenged mice as determined by *in vivo* imaging. Mice with RLU values lower than 4.10^4^ (absence of bioluminescent signal) were considered protected and mice with RLU above 1.10^5^ (clear bioluminescent signal) were considered unprotected. Mice showing ‘a weak spot’ of bioluminescence signal were excluded from the immunological analysis as outcome of protective immunity was doubtful. The parasite liver loads after primary immunization and boost were again comparable in IV-I and ID-I mice and after challenge, we again observed a comparable difference in protection as observed without artesunate treatment; i.e. 53% and 95% protection in ID-I and IV-I mice, respectively (Fig. 2). Because drug treatment itself may modulate immune responses^45, 46^ and thereby influence the detection of immunization-induced responses, we compared several hepatic immune parameters at day 7 after challenge of naïve and IV-I mice both with and without artesunate treatment. In protected IV-I mice comparable responses were detected for CD8 and CD4 memory T cell induction, T cell interferon (IFN)-γ expression, and B cell interleukin (IL)-10 expression, whereas a trend, though not significant, for lower frequencies of IL-10-expressing Foxp3^+^ regulatory T (Treg) cells in artesunate treated mice in both naïve and protected IV-I mice (Fig. S5). These results indicate that artesunate treatment can be applied for prevention of blood stage infection after challenge without affecting hepatic immune responses in experiments described below.

### Unprotected ID-I mice show reduced effector and higher regulatory hepatic immune responses compared to ID-I and IV-I protected mice

To explain a differential protection outcome despite a comparable parasite liver load, we hypothesized that protected and unprotected ID-I mice may display differences in hepatic immunity. We therefore studied the hepatic immune responses in ID-I mice treated with artesunate to exclude any interference of blood stage parasitemia in the unprotected mice. Subsequently, ID-I hepatic immune responses were compared to those found in IV-I mice.

#### Unprotected ID-I mice show less CD8 and CD4 T cell effector responses compared to protected mice but similar CSP-antibody titers

**Figure 3:**
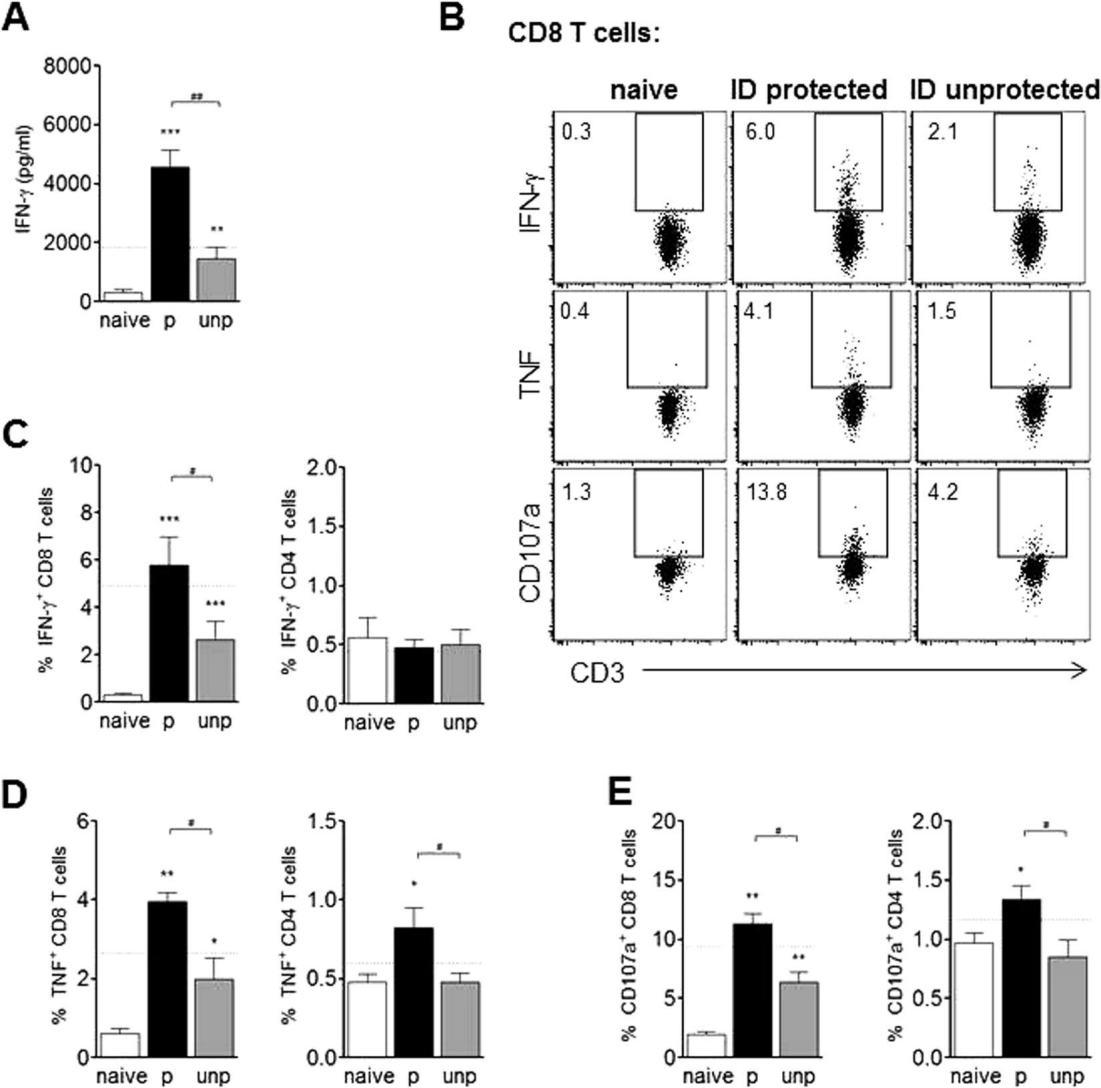
Unprotected mice after ID immunization show reduced liver T cell effector responses compared to protected mice. ID immunized mice were challenged followed by artesunate treatment, and distinguished as protected (p, i.e. luciferase negative) or unprotected (unp, i.e. luciferase positive). Hepatic CD8 and CD4 T cells were analyzed at day 7 after challenge. **(A)** IFN-γ concentration in supernatant of total leukocytes after culture for 36 h with CSP and sporozoites as measured by ELISA. **(B)** Representative FACS plots of CD8^+^ gated T cells for intracellular expression of IFN-γ or TNF after culture for 4 h with CSP and brefeldin A, and for surface expression of CD107a after 4 h culture with CSP, brefeldin A and monensin. Numbers indicate the frequency of the gated cell population. Graphs show a summary of 2 experiments with 8–14 mice per group for frequency of IFN-γ (**C**), TNF (**D**), or CD107a (**E**) -expressing CD8 T cells and CD4 T cells. The dotted line indicates the mean cytokine or expression level for protected IV immunized mice (N = 9–16). Significant difference by Mann-Whitney test is indicated by *p < 0.05, **p < 0.01, ***p < 0.001 (to naïve control group), and ^#^p < 0.05, ^##^p < 0.01 (between immunized mouse groups).

T cell effector responses are crucial for protection in human and animal models^21, 47–51^ and might therefore explain the differential protection outcome. At day 7 after challenge, IFN-γ secretion of total hepatic leukocytes in response to ‘circumsporozoite protein’ peptide (CSP) and sporozoites was indeed 3.1-fold higher in protected compared to unprotected ID-I mice (Fig. 3A). Intracellular flow cytometric analysis revealed that CD8 T cells were the main source for CSP-specific IFN-γ, showing significantly more IFN-γ in protected compared to unprotected ID-I mice (Fig. 3B,C), which was similar to levels found in protected IV-I animals (indicated by dotted line). In CD4 T cells, IFN-γ was not even upregulated compared to naïve mice which could be due to insufficient restimulation by CSP peptide, as opposed to polyclonal stimulation (Fig. S6B). Furthermore, the frequency of CD8 T cells expressing tumor necrosis factor alpha (TNF) and surface-exposed CD107a (a surrogate marker for cytotoxic activity) was higher in protected ID-I compared to unprotected ID-I mice (Fig. 3D,E), while expression of the cytolytic enzyme granzyme B in CD8 T cells did not differ between groups (Fig. S6A). As for CD8 T cells, also the CD4 T cell population showed a higher TNF and CD107a expression in protected ID-I mice (Fig. 3D,E), Interestingly, when applying a polyclonal stimulation by PMA/ionomycin, IFN-γ and TNF production by CD8 and CD4 T cells were largely similar between protected and unprotected ID-I animals (Fig. S6B,C), suggesting specific differences for *Plasmodium*-specific T cell responses only. Next, we compared the induction of memory T cells in protected and unprotected ID-I animals. The frequency of total CD44^+^ (memory) T cells in livers of unprotected mice was equal (CD8 T cells) or even higher (CD4 T cells) compared to protected ID-I mice (Fig. S6D). However, the CD8 memory T cell frequencies (mean 41.2%) were significantly less compared to those of protected IV-I mice (50.8%, indicated by dotted line) pointing to a different capacity to induce T cell memory by different routes of immunization. Next to hepatic T cell responses, we checked for differences in peripheral blood mononuclear cells (PBMC) between protected and unprotected ID-I mice and similar for the livers, we observed that T cell effector responses in peripheral blood were stronger upregulated in protected mice than unprotected mice compared to naïve controls (Fig. S7).

Next to cellular components, antibody responses have been associated with protection by whole (attenuated) sporozoite immunization and increased anti-CSP titers were shown to reduce liver parasite burden in a dose-dependent manner^52, 53^. However, we did not find significant differences in CSP-induced plasma antibody titers between protected or unprotected mice (Fig. S8), indicating that CSP-specific antibodies do not play a role in the observed difference in protection after ID and IV-I.

Taken together, despite similar hepatic parasite loads during immunization, unprotected mice developed mostly weaker CD4 and CD8 T cell effector responses compared to protected mice in both liver and peripheral blood, while CSP antibody titers were comparable.

#### Unprotected ID-I mice have higher regulatory immune responses compared to protected mice

**Figure 4:**
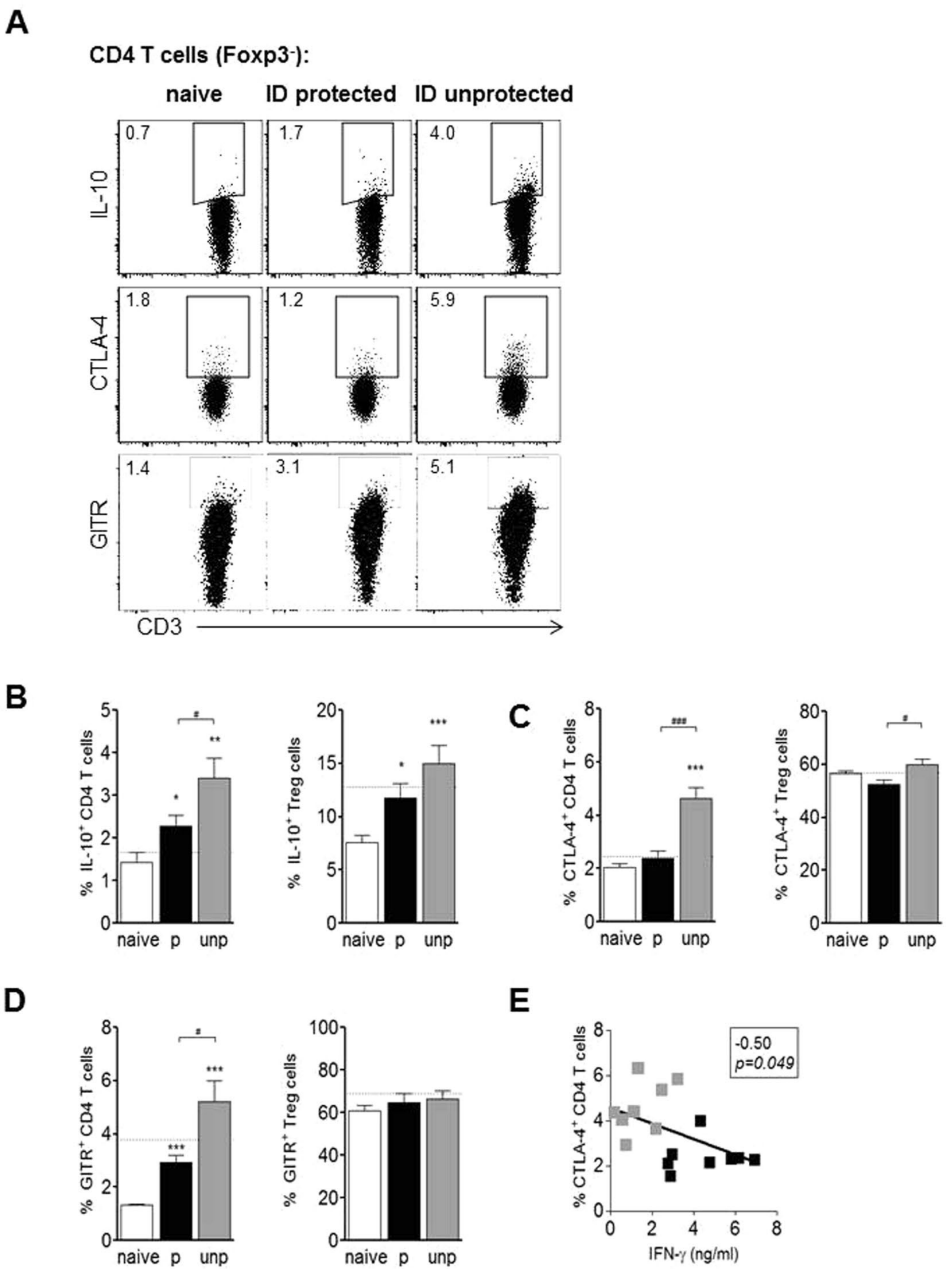
Unprotected mice have higher regulatory immune responses compared to protected mice. Hepatic CD4 T cells (gated Foxp3^−^) and Foxp3^+^CD25^+^ Treg cells of protected and unprotected ID immunized mice were analyzed for regulatory marker expression at day 7 after challenge under artesunate treatment. **(A)** Representative FACS plots of Foxp3^−^ gated CD4 T cells for intracellular IL-10 expression after 36 h culture of hepatic leukocytes with CSP and sporozoites and addition of PMA/ionomycin plus brefeldin A in the last 4 h, and for surface expression of CTLA-4 or GITR *ex vivo*. Graphs show a summary of **(B)** intracellular IL-10 expression, **(C)** surface CTLA-4 and **(D)** GITR expression of Foxp3^−^ CD4 T cells and Treg cells from 2 experiments with 8–14 mice per group. The dotted line indicates the mean frequency for protected IV immunized mice (N = 9–16). Significant difference by Mann-Whitney test is indicated by *p < 0.05, **p < 0.01, ***p < 0.001 (to naïve control group), and ^#^p < 0.05, ^###^p < 0.001 (between immunized mouse groups). **(E)** The production of secreted IFN-γ by hepatic leukocytes was correlated with the percentage of hepatic CTLA4^+^ Foxp3^−^ CD4 T cells. Correlation coefficient and p-value are indicated.

Regulatory T cell responses can suppress the development of pro-inflammatory T cell responses^54, 55^ and might provide an explanation for the observed lower T cell effector response and suboptimal protection efficacy by ID immunization. However, hepatic Treg cell frequencies, cell numbers, and intracellular IL-10 expression upon restimulation with CSP and sporozoites were comparable between protected and unprotected ID-I mice (Fig. 4B and Fig. S9A,B). Next to Treg cells, also Foxp3-negative CD4 T cells can fulfill regulatory function^56^. Indeed, unprotected mice had a significantly higher frequency of IL-10-expressing Foxp3-negative CD4 T cells compared to protected ID-I mice (3.4% vs. 2.3%) (Fig. 4A,B). Interestingly, the IL-10 expression of CD4 T cells from protected ID-I animals was still significantly higher compared to protected IV-I mice (in average 2.3% vs. 1.7%), and the same held true for B cell IL-10 expression (2.9% vs. 2.0%; Fig. S8C), supporting the idea of a generally higher IL-10 induction after ID immunization compared to the IV route. Also the regulatory markers CTLA-4 and GITR were significantly higher expressed on Foxp3-negative CD4 T cells of unprotected ID-I mice compared to protected ID-I mice, while Treg cells showed only a small but significantly higher expression for CTLA-4 but not GITR (Fig. 4C,D). Additionally IFN-γ secretion of total leukocytes negatively correlated with CTLA-4 (correlation coefficient −0.5 and *p* = *0.049*; Fig. 4E). Collectively, these data indicate a stronger hepatic regulatory immune response being induced in unprotected ID-I animals which inversely correlated with a reduced T cell effector response.

### ID immunization induces less effector and more regulatory hepatic immune responses compared to IV immunization during the immunization schedule

Using the IV and ID immunization protocol that resulted in similar liver loads, we found a suboptimal protection by the ID route compared to IV route that was associated with a suboptimal induction of effector immune responses together with increased regulatory responses in livers of unprotected ID-I mice. These responses were measured 7 days after challenge and we wondered whether the differences in immunity by the different routes of immunization were already induced at an earlier stage, i.e. after the primary immunization or after boost immunization.

#### ID-I mice have less activated hepatic CD44^+^ CD8 T cells compared to IV-I mice

**Figure 5:**
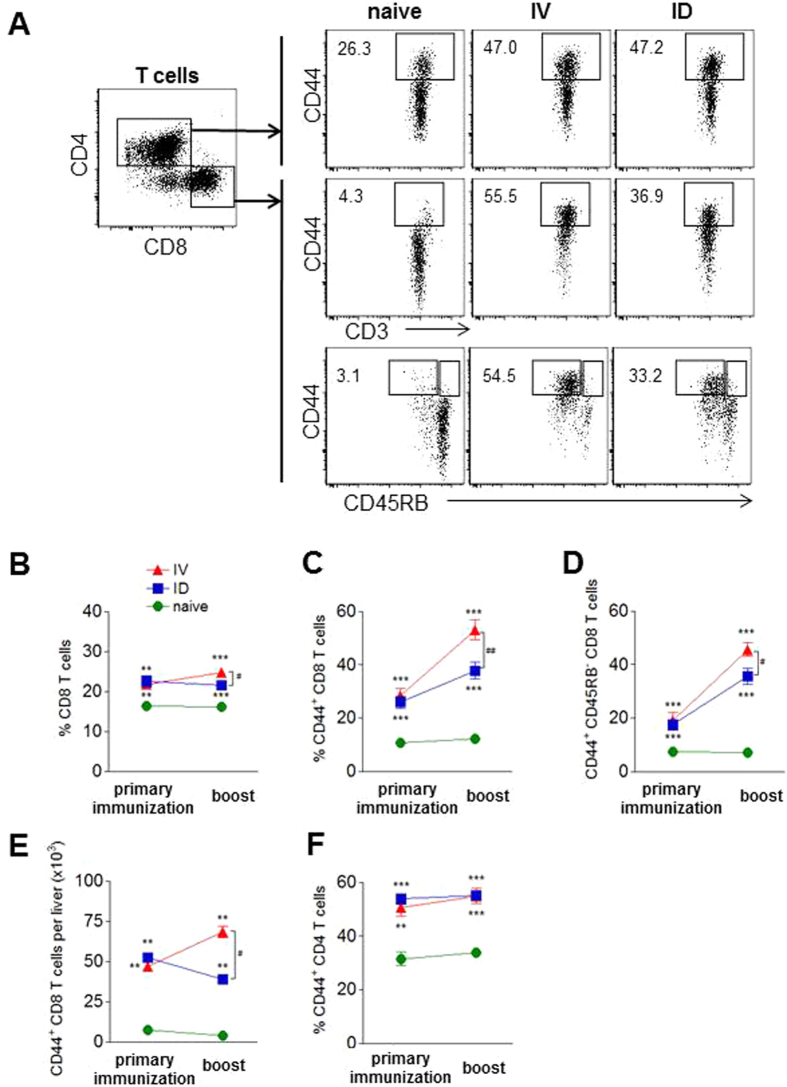
Induction of activated CD44^+^ CD8 T cells is less by ID compared to IV immunization. BALB/c mice were immunized twice 2 weeks apart with liver-attenuated luciferase-expressing ΔPyFabBF-GFP-Luc_con_ sporozoites (PyGAP) either with 10 K IV or 50 K ID. Expression of the activation and memory markers CD44 and CD45RB on hepatic T cells at day 7 after primary immunization or boost were analyzed by flow cytometry. **(A)** Representative FACS plots of CD8^+^ and CD4^+^ gated T cells after IV or ID boost compared to a naïve control. Numbers indicate the frequency of the gated cell population. **(B)** Frequencies of CD8^+^ T cells within total CD3^+^ T cells, of **(C)** CD44^hi^ cells and **(D)** CD45RB^low^ CD44^hi^ cells within the CD8 T cell population, **(E)** number of CD44^hi^ CD8 T cells per liver, and **(F)** of CD44^hi^ cells within the CD4 T cell population. Summary of 2 experiments with 8–10 mice per group. Significant difference by Mann-Whitney test is indicated by **p < 0.01, ***p < 0.001 (to naïve control group), and ^##^p < 0.05, ^##^p < 0.01 (between immunized mouse groups).

A fast activation of T cells in response to antigen during immunization with whole sporozoites is a critical step to achieve protection^32, 47, 48, 57–59^. Therefore, expression of effector molecules and the T cell activation marker CD44 was analyzed 7 days after IV or ID primary immunization and after boost immunization, without subsequent challenge. A significant increase was observed of the total CD8 T cell population and the frequency of activated or memory CD44^+^ and CD44^+^ CD45RB^+^ CD8 T cell subsets within hepatic leukocytes compared to naïve controls. However, after the boost, this increase was significantly smaller in ID-I mice compared to IV-I mice, which is also reflected by a lower number of CD44^+^ CD8 T cells per liver (38% or 38.9 × 10^3^ CD44^+^ CD8 T cells compared to 53% or 68.3 × 10^3^; Fig. 5A–E; Table S1). CD44^+^ CD4 T cell frequencies were equally upregulated in IV-I and ID-I mice (Fig. 5F). Next, we evaluated CD8 T cell effector functions. Upon restimulation by PMA/ionomycin, IFN-γ expression was significantly higher in IV-I mice after boosting compared to ID-I mice (Fig. S10A), but CSP-specific IFN-γ and cytolytic enzyme granzyme B expression were equally upregulated in both IV-I and ID-I mice (Fig. S10B,C).

Because of the lower increase in activated T cells in ID-I mice after boosting we tested for T cell exhaustion during ID-I as an explanation for the observed suboptimal protection rates. The major inhibitory receptor regulating T cell exhaustion, programmed cell death-1 (PD-1), is known to play a role in a strong reduction of CD8 T cell numbers (and function) during malaria infections^60^. In our ID-I and IV-I mice, expression of PD-1 was to a similar extent upregulated on both CD8 T cells and CD4 T(reg) cells compared to naïve controls (Fig. S11A–C). The ligand PD-L1 was even higher expressed on CD11c^hi^MHCII^+^ conventional DCs in IV-I mice compared to ID-I mice (Fig. S11D). These observations indicate that a difference in T cell exhaustion between ID and IV immunizations does not account for the observed differences in the level of protection. Collectively, ID-I induced equally high parasite-specific CD8 T cell effector functions in the liver, but resulted in less activated CD8 T cell subset formation compared to IV-I, which is unlikely to be caused by differences in T cell exhaustion.

#### CD8 dendritic cell subset frequencies are lower in ID-I mice compared to IV-I mice

**Figure 6:**
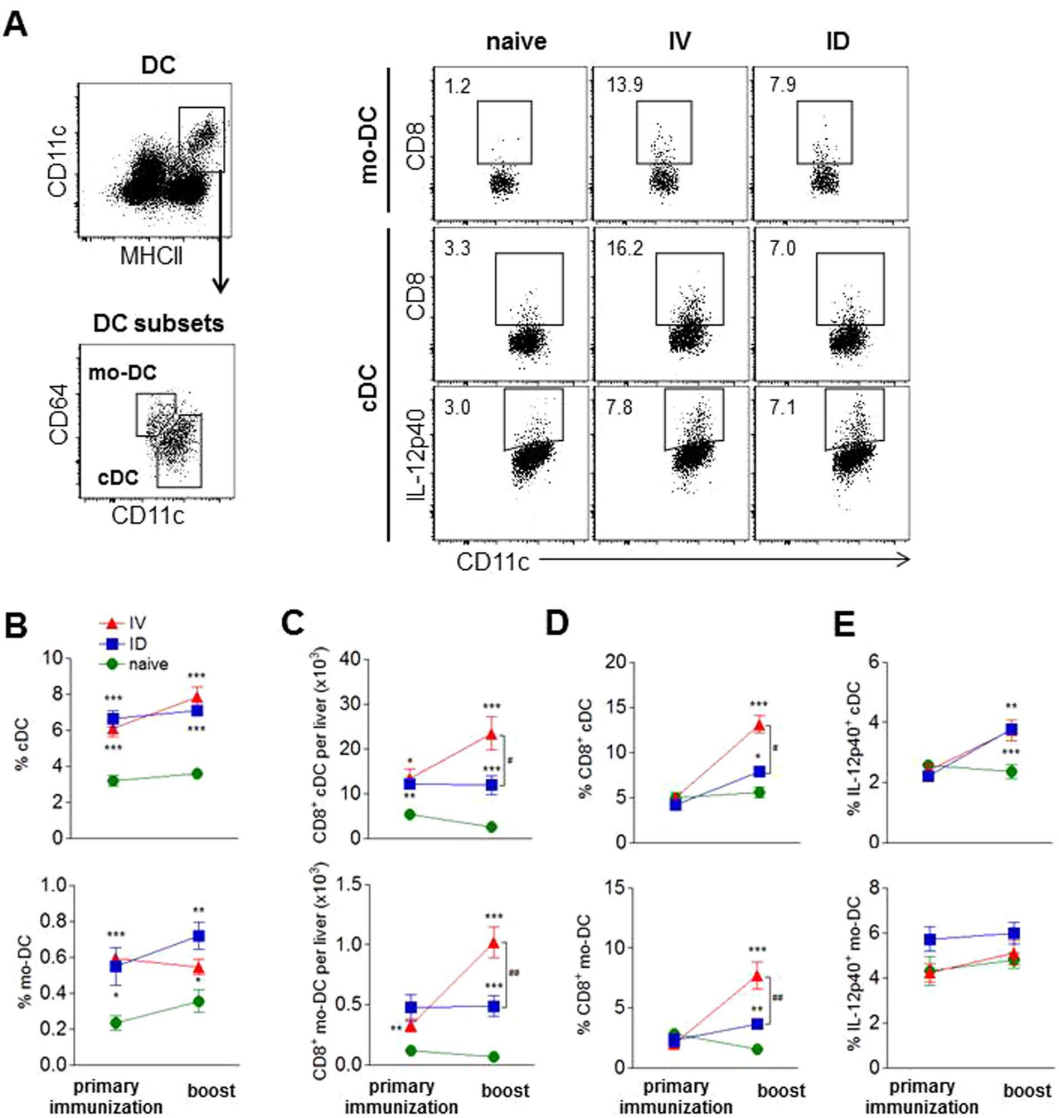
CD8 dendritic cell subset frequencies are smaller after ID compared to IV immunization. Dendritic cell (DC) subsets in liver were analyzed by flow cytometry at 7 days after primary or boost immunization via IV or ID route. **(A)** MHCII^+^CD11c^hi/int^ DC were gated for CD11c^int^CD64^hi^ monocyte-derived DC (mo-DC) and CD11c^hi^CD64^low^ conventional DC (cDC) after excluding F4/80-expressing macrophages. Representative FACS plots of both DC subsets for CD8 and intracellular IL-12p40 expression in mice 7 days after IV or ID boost or in naïve control mice are shown. Numbers indicate the frequency of the gated cell population. **(B)** Frequencies of DC subsets within total leukocytes. **(C)** Number of CD8-expressing DC per liver. **(D)** Frequencies of CD8-expressing DC within the mo-DC or cDC subset. **(E)** Intracellular IL-12p40 expression of DC subsets after culture of leukocytes with brefeldin A for 4 h. Summary of 2 experiments with 8–10 mice per group. Significant difference by Mann-Whitney test is indicated by *p < 0.05, **p < 0.01, ***p < 0.001 (to naïve control group), and ^#^p < 0.05, ^##^p < 0.01 (between immunized mouse groups).

Suboptimal CD8 T cell activation may result from suboptimal priming by CD8^+^ dendritic cells (DC)^34^. Earlier studies showed that IV-I leads to accumulation of CD8^+^ DC in liver with a highly developed T cell memory inducing capacity^34, 61^. We therefore analyzed both conventional DC (cDC) and monocyte-derived DC (mo-DC) populations in liver after IV or ID primary immunization and boost immunization to investigate whether differences in these cell populations could explain the lower CD8 T cell activation observed in ID-I animals. Both after the primary and boost immunization the frequency of CD11c^hi^MHCII^+^ cDC as well as CD11c^int^MHCII^+^CD64^hi^ mo-DC within hepatic leukocytes was similarly increased in IV-I and ID-I mice compared to naïve controls (Fig. 6B). Although a similar increase was found in the number of CD8^+^ cDC and CD8^+^ mo-DC after the primary immunization in both ID-I and IV-I mice compared to naïve controls, these numbers were only further increased after boosting in the IV-I mice resulting in lower CD8^+^ DC frequencies and numbers in ID-I mice compared to IV-I mice (Fig. 6C,D). In contrast to the DC subset composition, there was no difference with respect to intracellular IL-12p40 expression, which was similarly upregulated in cDC after boost in both IV-I and ID-I mice (Fig. 6E). Combined, these data indicate a suboptimal recruitment of CD8^+^ DC subsets in the liver after ID immunization which might be linked to the suboptimal activation of CD8 T cells.

#### ID-I induces stronger regulatory immune responses in the liver compared to IV-I

**Figure 7:**
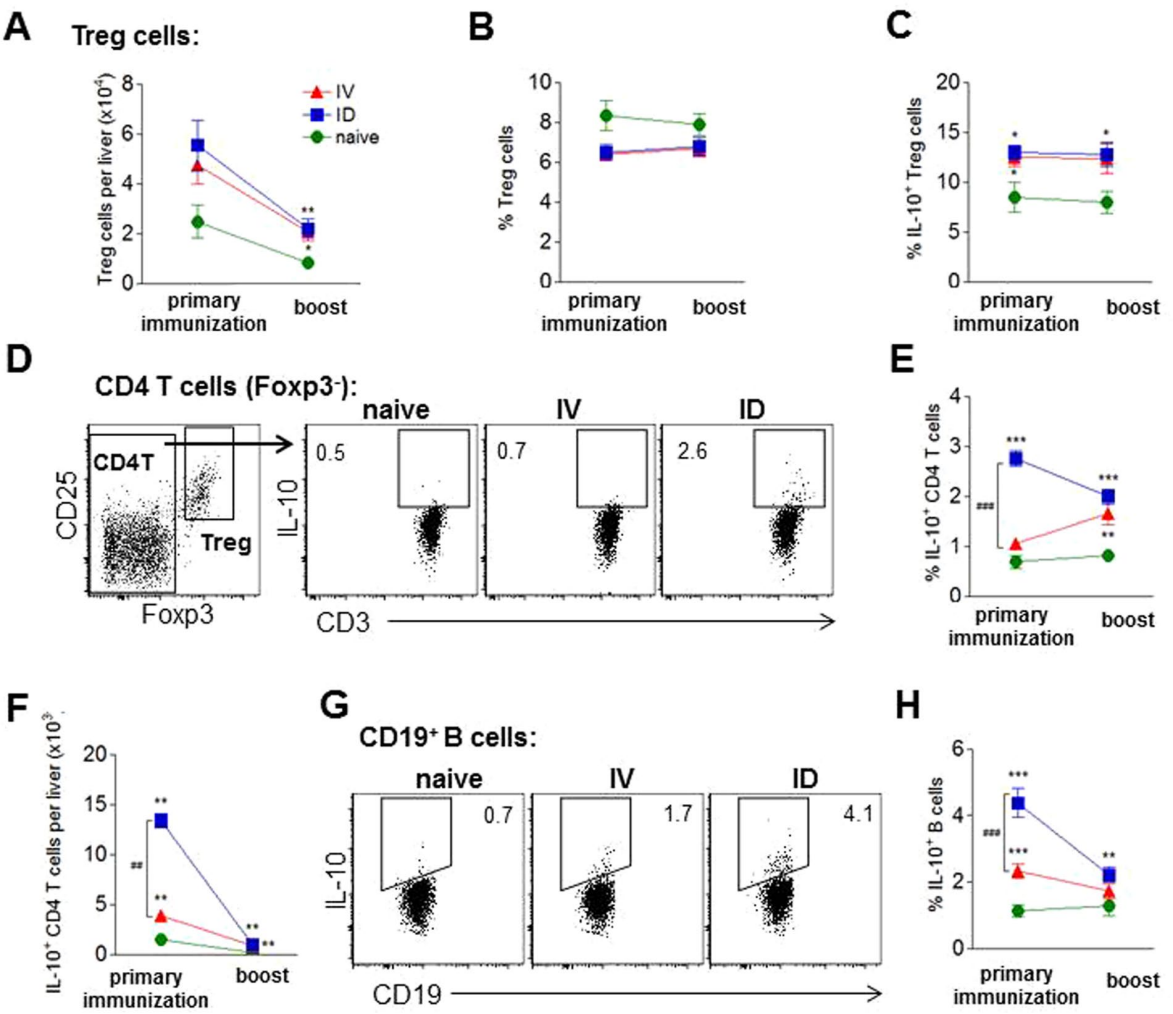
ID immunization induces stronger regulatory immune responses in liver compared to IV. Hepatic leukocytes were analyzed 7 days after IV or ID primary immunization or boost for regulatory marker expression by flow cytometry directly *ex vivo* (**A**,**B**) or after 36 h culture with CSP and sporozoites (**C**–**G**). **(A)** Number of Foxp3^+^CD25^+^ Treg cells per liver. **(B)** Frequency of Treg cells within the CD4^+^ T cell population. **(C)** Intracellular IL-10 expression of Treg cells after addition of PMA, ionomycin and brefeldin A for 4 h to the culture. **(D)** Representative FACS plots of CD4^+^ Foxp3-negative T cells (CD4 T) in one IV or ID immunized and a naïve control mouse. **(E,F)** Summary of intracellular IL-10 expression of CD4^+^Foxp3^−^ T cells after addition of PMA, ionomycin and brefeldin A expressed as frequencies (**E**) and cell number per liver (**F**). Representative FACS plots (**G**) and summary (**H**) of intracellular IL-10 expression in CD19^+^ gated B cells after addition of PMA, ionomycin and brefeldin A. Graphs show 1 representative out of 2 similar experiments (**C**) or a summary of 2 experiments with 8–10 mice per group. Significant difference by Mann-Whitney test is indicated by *p < 0.05, **p < 0.01, ***p < 0.001 (to naïve control group), and ^#^p < 0.05, ^##^p < 0.01 (between immunized mouse groups).

Because we had observed a higher IL-10 production in both B and T cells after challenge in ID-I mice, we next investigated whether regulatory responses are already upregulated after primary immunization and boost immunization which may explain the differences in DC and activated T cell subset induction observed between IV-I and ID-I mice. To this end, hepatic leukocytes were collected 7 days after IV or ID primary and boost immunization and analyzed for regulatory marker expression. Similar as observed after challenge, the frequency, number and CSP-specific IL-10 and PD-1 expression of Foxp3^+^CD25^+^ Treg cells was equal in livers of IV-I and ID-I mice (Fig. 7A–C; Fig. S10C), making it unlikely that differences in number and activation of Foxp3^+^ Treg cells can explain the observed differences between ID and IV immunization. In contrast to Treg cells and similar to what was found after challenge, the frequency and cell number per liver of IL-10-expressing Foxp3-negative CD4 T cells was significantly higher in ID-I mice after primary immunization compared to IV-I mice (in average 2.8% or 13.4 × 10^3^ versus 1.0% or 3.9 × 10^3^ IL-10^+^ cells; Fig. 7D–F; Table S1). Also the number of CTLA-4-expressing CD4 T cells was higher in ID-I mice (Table S1), although this was not reflected by cell frequencies which were similarly increased by both immunization routes as it was the case for GITR (Fig. S12A,B). To draw conclusions on the nature of IL-10-expressing Foxp3-negative T cells, we performed a double-staining for IL-10 and IFN-γ (Fig. S12C). The results show that the frequencies of both IL-10-producing subsets, IL-10/IFN-γ double positive and IL-10 single positive, are significantly higher in ID compared to IV immunized animals (Fig. S12D). Both subsets are similar abundant in ID animals, which suggests that different types of regulatory IL-10-expressing CD4 T cells are present, amongst which the type 1 regulatory (Tr1) cells which are characterized by IL-10 but no or very low IFN-γ expression^56^.

Interestingly, B cells showed a similarly increased IL-10 expression as Foxp3-negative T cells in ID-I mice after primary immunization compared to IV-I mice (2.2-fold higher frequency; 20.4 × 10^3^ cells per liver compared to 13.2 × 10^3^), which returned to baseline levels after the boost immunization in both groups (Fig. 7G,H; Table S1). Thus, ID-I led to a higher IL-10 production in CD4 T cells and B cells, compared to IV-I. Interestingly, this was particularly evident after the primary immunization, preceding the suboptimal DC and T cell subset induction which was only observed after the boost immunization.

#### Lymph node regulatory responses are increased by ID-I compared to IV-I

**Figure 8:**
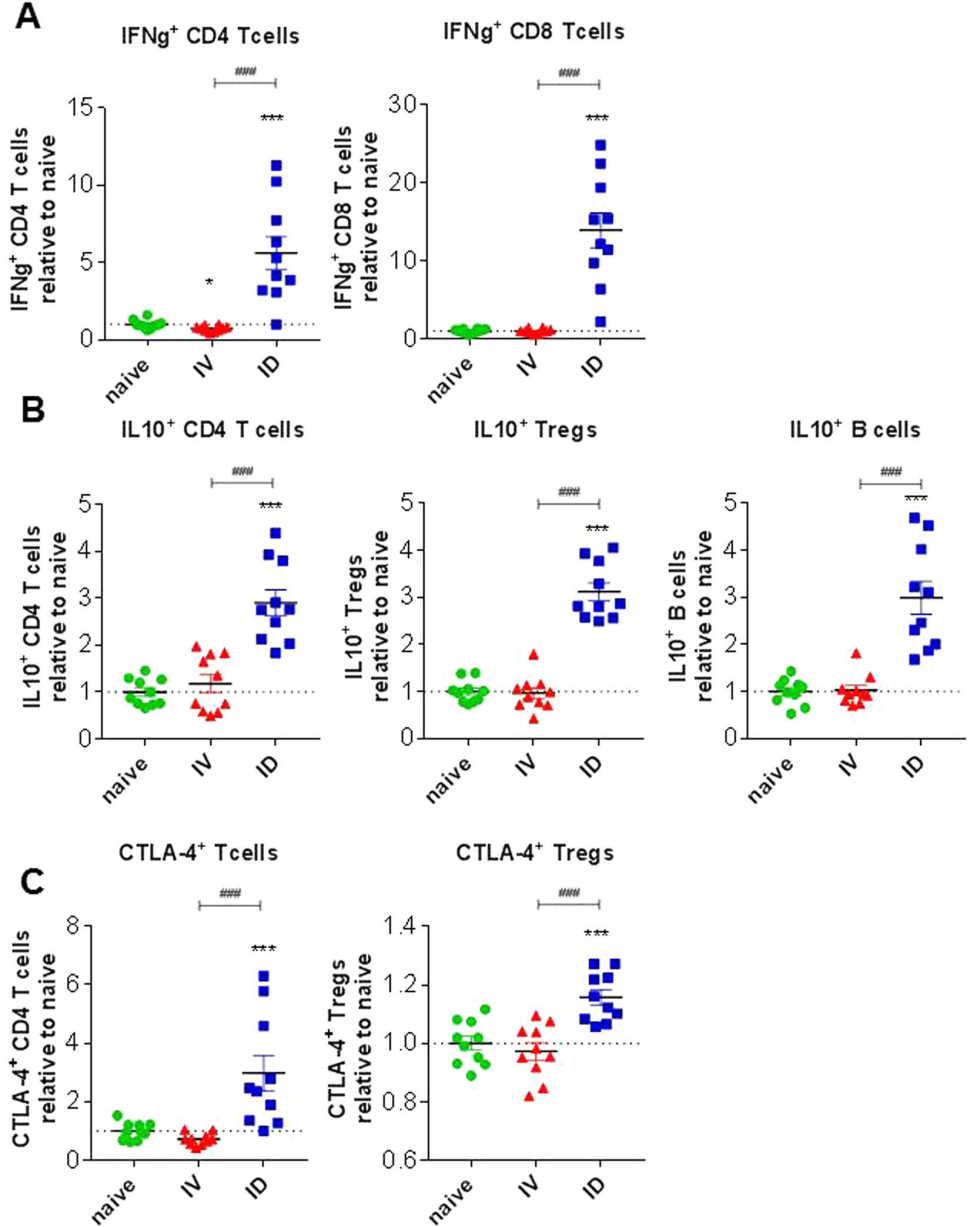
ID but not IV immunization increases regulatory immune responses in the skin-draining lymph node. Inguinal lymph node cells were analyzed 7 days after IV or ID primary immunization for regulatory marker and cytokine expression. Flow cytometry was performed after 36 h culture with CSP and sporozoites and addition of PMA, ionomycin and brefeldin A in the last 4 h (**A**,**B**), or directly *ex vivo* (**C**). Frequencies calculated relative to naïve control are given for **(A)** IFN-γ^+^ CD4 T cells and CD8 T cells, **(B)** IL-10^+^ Foxp3^−^ CD4 T cells, IL-10^+^ Foxp3^+^CD25^+^ Treg cells, IL-10^+^ CD19^+^ B cells, and **(C)** CTLA-4^+^ Foxp3^−^ CD4 T cells and CTLA-4^+^ Foxp3^+^CD25^+^ Treg cells. Summary of 2 experiments with 10 mice per group. Significant difference by unpaired t-test is indicated by *p < 0.05, **p < 0.01, ***p < 0.001 (to naïve control group), and ^##^p < 0.01, ^###^p < 0.001 (between immunized mouse groups).

After ID immunization, the skin-draining lymph node is one of the first sites of immunological responses, and may influence protection outcome or shape down-stream immunity in liver. Because we had observed increased regulatory responses in liver 7 days after primary ID-I, we investigated whether the same holds true for cells of the inguinal lymph node draining at the site of sporozoite administration (Table S2). We find increased frequencies of IFN-γ producing CD4^+^ and CD8^+^ T cells in ID-I compared to IV-I mice (Fig. 8A). However, in addition we find significantly increased frequencies and cell numbers of several IL-10-expressing cell types in lymph nodes (B cells, Foxp3^−^ CD4 T cells, Foxp3^+^ Treg cells) (Fig. 8B). The same was true for the frequency of CTLA-4-expressing Foxp3- CD4 T cells (Fig. 8C). Thus, next to IFN-γ producing T cells, ID-I also substantially increases regulatory responses in both, liver and skin-draining lymph nodes.

Taken together, ID-I not only induced suboptimal protection compared to IV immunization, but also led to increased frequencies of IL-10 producing B and T cells in lymph nodes and liver as well as to suboptimal hepatic CD8 (memory) T cell immune responses, a defect already detected during immunization in ID-I mice and which remained detectable after challenge in the group of unprotected ID-I mice. Importantly, these differences in protective efficacy and protective immunity developed despite comparable parasite livers load between both immunization routes.

## Discussion

A prerequisite for induction of protective immunity by immunization with injected sporozoites is that they are alive and retain the capacity to invade hepatocytes. In both human and mouse studies the number of sporozoites administered correlates with the level of protective immunity, and in mouse models the level of parasite liver loads associates with the level of protection. Based on these observations, it has been assumed that the parasite liver load after immunization is the most critical factor in determination the level of protective immunity.

In contrast to what has been suggested in previous studies we found that differences in protection outcome and hepatic immune response between IV and ID immunization are not exclusively explained by differences in liver load. Our data provide new insights on the immunization-induced activation and recruitment of specific cell subsets into liver, an understudied organ in terms of immunological consequences of sporozoite immunization. This involves, amongst others, antigen-specific CD8 T cell responses, mo-DC as well as IL-10-producing T and B cells as likely critical determinants in protection outcome.

It is generally accepted that CD8 T cells play a central role in protective immunity induced by immunization with live sporozoites in rodent malaria models. Our data support this important role as unprotected mice had weaker antigen-specific CD8 T cell effector responses both in liver and peripheral blood. While we could not detect differences in IFN-γ expression by CD8 T cells induced by the different routes of immunization (Figs 3C, S10B), we found higher frequencies of CD8 T cells and CD8 memory T cells in IV-I mice compared ID-I. This could be explained by an increased CD8 T cell division which might play a role for protection outcome in our immunization/boost/challenge model. Similarly, in a recently published study applying adoptive transfer of *Plasmodium*-specific CD8 T cells prior to IV or ID injection of sporozoites, IV led to higher cell division of antigen-specific CD8 T cells in liver and hepatic lymph nodes than ID administration^62^. With respect to CD4 T cells, recent observations in individuals vaccinated with RTS-S, a recombinant CSP-based vaccine, and in individuals naturally exposed to malaria suggest an important role for CD4 T cell production of TNF, with or without IFN-γ, as a potential immune correlate of protection^63^. This is in line with our findings where CD4 T cell expression of TNF was only induced in protected but not unprotected animals.

Next to T cells, also CD8^+^ DC were found to accumulate in liver after IV immunization with radiation-attenuated sporozoites and these cells were highly potent in driving development of CD8 memory T cells *ex vivo* and to confer protection upon adoptive transfer^61, 64^. Also in our IV-I mice the frequency of CD8^+^ cDC increased from low steady-state frequencies up to about 15% within the total DC population after boosting, a similar level as obtained after immunization with radiation-attenuated sporozoites^61^. Interestingly, after boosting both CD8 DC and CD44^+^ CD8 T cell subset frequencies were significantly lower in ID-I mice compared to IV-I animals, suggesting a positive correlation between low DC and T cell responses and weak development of protective immunity by ID immunization. However, it is well established that T cells can also be primed by DCs within the skin-draining lymph node and then recirculate to the liver, at least after exposure to sporozoites through infected mosquito bites^65^. Although mosquito-bite is not exactly comparable to ID administration, a similar phenomenon of DC priming in skin-draining lymph node might occur as well after ID immunization. Thus, while we found several significant differences in liver for both DC and T cell subsets between ID and IV immunization which might be related to differences in the level of protection, it is still unclear where T cell priming by DC occurs.

In contrast to conventional DC, surprisingly little is known about the role of monocyte-derived DC during malaria infection and immunization. We provide the first indication that mo-DC accumulate in the liver and that the extent of accumulation is influenced by the immunization route. Also in other protozoan or viral infection models, mo-DC were shown to accumulate at the site of infection and contribute to CD4 T cell and CD8 memory T cell induction^66, 67^. This is in line with the concept of local DC differentiation, in which circulating precursors give rise to DC subsets in peripheral organs. Interestingly, in studies of microbial and viral infections it was found that mo-DC efficiently cross-present antigen to CD8 T cells, that T cell priming can be even more powerful than by conventional CD8^+^ DC and that T cell priming was enhanced by linking TLR agonists to an antigen^68, 69^. These observations warrants further studies on malaria-associated mo-DC and poses novel opportunities to improve malaria vaccine efficacy by designing mo-DC-enhancing antigens or adjuvants as part of a general strategy to support vaccination against infectious diseases^70^.

Several studies have described induction of regulatory responses during malaria infections in various organs by malaria infected red blood cells (iRBCs). Improved clearance of iRBCs has been observed in the absence of Treg-derived IL-10^71, 72^. Only few studies have addressed regulatory cells after immunization^71, 73, 74^. Unfortunately, those studies do not provide insight whether the observed regulatory responses are only a consequence of blood stage infections or can already be induced by the pre-erythrocytic stages, i.e. the sporozoites and the liver stages. To rule out the effect of blood stage infection and iRBCs on the induction of regulatory responses we treated immunized mice after challenge with wt parasites with artesunate to prevent the development of blood stage infections in unprotected mice. We found increased regulatory responses in the liver of unprotected ID-I mice, which show that regulatory responses in these mice were induced without blood-stage infections. We demonstrated that after wt challenge unprotected mice have higher frequencies of activated Foxp3^+^ Treg cells in liver, showing increased CTLA-4 expression compared to protected mice. In mice with *P. yoelii* blood stage infections another IL-10 producing but Foxp3-negative regulatory T cell type termed Tr1 cells was found to suppress clearance of iRBC^71, 75^. Here, we found that also in the absence of blood stages unprotected ID-I mice have higher frequencies of Foxp3-negative CD4 T cells with regulatory phenotype (IL-10, CTLA-4, GITR) compared to those cells in protected ID-I mice, and this expression was negatively correlated with IFN-γ expression, suggesting the induction of regulatory and suppression of proinflammatory immune responses by pre-erythrocytic stages. Our data thus indicate that the ratio of pro- versus anti-inflammatory markers (such as IFN-γ, TNF vs IL-10, CTLA4) associates with control of liver infection after immunization. This might be true also for humans in which control of blood stage infection associated with the ratio of TNF versus IL-10^76–78^.

Another important player in the network of regulatory cells are regulatory B (Breg) cells. Despite the attention that Breg cells have attracted in studies of other infectious or inflammatory diseases, including protozoan infections^79, 80^, Breg cells have so far largely been understudied in studies of malaria infections. Regulatory B cells usually express high levels of IL-10 and have been linked to immune responses in different disease states, such as regulation of auto-immunity, allergic disease and helminth infections^81–83^. As far as we know, only one study has addressed the induction of IL-10 producing B cells during malaria infections, in which these cells were detected in spleens of mice with *P. berghei* blood stage infection and were associated with controlling cerebral pathology^84^. Our study provides the first evidence that IL-10 producing B cells are also induced in skin-draining lymph node and liver upon immunization with sporozoites, and our observations show that ID immunization leads to stronger induction of these cells compared to IV immunization, very similar to what we observed for IL-10 producing Foxp3-negative T cells.

An important question is how the elevated frequencies of different IL-10 producing cell types we observed in this study might affect immunity in ID-I mice. It has been recently described that IL-10 promotes the maturation of memory CD8 T cells during bacterial and viral infection^85, 86^, while other studies showed that IL-10 suppressed memory induction in acute bacterial, viral and parasitic infections^85–87^. We observed a weaker induction of CD44^+^ CD8 (memory) T cells in ID-I mice compared to IV-I mice, both after boosting and after challenge. Interestingly, IL-10 induction occurred already after primary immunization and thus preceded the suboptimal DC and memory T cell subset induction in ID-I mice. In particular, we found IL-10-expressing CD4 T cells with characteristics of type 1 regulatory (Tr1) T cells (Foxp3 negative, high IL-10, no/low IFN-γ expression). This antigen-specific subset is known to be induced by antigen exposure, such as during infection or immunization, from naïve CD4 T cells in the periphery, and to subsequently suppress Th1 responses^56^. This subset might be pivotal in suppressing malaria protective immunity in our model, as was described for other protozoan infections^88^. Furthermore, we found regulatory and effector responses to negatively correlate with each other after challenge of the mice. Because we could not find support for alternative explanations of the low protective efficacy of ID immunization, such as increased T cell exhaustion or low parasite-specific antibody titers, it is tempting to speculate that regulatory immune cells play a dominant role in suppressing the development of DC and effector T cell function.

ID immunization apparently favors development of IL-10 expression in liver, as frequencies of IL-10 expressing CD4 T cells and B cells were higher in ID-I mice already after primary immunization compared to IV-I mice. This might indicate that certain immune evasion mechanisms are initiated by ID administered sporozoites. IV administered sporozoites reach the liver within minutes^89^, whereas it is known that most ID administered sporozoites remain in the skin for hours before entering the bloodstream and invading a hepatocyte^90^. Sporozoites remaining in the skin at the site of administration might be associated with the development of regulatory responses which we found in skin-draining lymph nodes of ID-I mice, such as increased IL-10 expression by CD4 T cells next to IFN-γ producing T cells, suggesting the existence of a balance between regulators and effectors, which may boil down into differential migration or activation of hepatic responses, explaining why some ID-I mice are still protected and others not. For instance, local high levels of IL-10 at the site of sporozoite administration might contribute to a reduced induction of cytotoxic T cell responses by affecting function of dermal DC, a subset which has been described as principal migratory DC and being capable of efficiently presenting *Plasmodium* antigens and activating CD8 T cells in dermal lymph nodes^91^. Furthermore, cytotoxic T lymphocyte responses induced by transcutaneous immunization in a tumor model were counter-regulated by both regulatory T cells and IL-10^92^. The concept of regulatory immune responses being induced by skin-administered malaria vaccines is not new. It was proposed that regulatory skin responses might subsequently suppress protective liver immunity^93^. Expanding this concept, our data now suggest that regulatory cell types are not only induced in the skin as the site of vaccine administration, but also in the skin-draining lymph node and, importantly, in the liver itself. If regulatory cell types, in skin, lymph node or liver, are indeed contributing to the low protection by ID immunization needs to be addressed in future studies. This is the more important, as also in volunteers that were immunized with attenuated sporozoites^94, 95^, unprotected individuals displayed increased Treg cell proliferation in peripheral blood already after boost immunization. Although these activated regulatory cell types differ from those found in our study, these observations indicate an adverse role of activation of regulatory cells for protective immunity.

Dissecting regulatory responses will be essential to improve the protective immunity induced ID immunization. Only increasing the sporozoite immunization dose in ID-vaccination approaches may not be sufficient for obtaining levels of protection that are comparable to IV-vaccination with attenuated sporozoites. Novel (trans)dermal delivery techniques and adjuvants may need to be explored. Examples of adjuvants that are known to increase ID vaccine immunogenicity are synthetic immunostimulatory agents (like cytosine phosphoguanosine, CpG) in Hepatitis B vaccination^96^ or the application of imiquimod (Aldara cream), a synthetic toll-like receptor agonist which stimulates pro-inflammatory cytokine production and innate immunity at site of administration in influenza vaccination^97^. Although regulatory responses have not been analyzed in these studies, it is attractive to speculate on the capacity of these adjuvants to overcome immunoregulatory processes, leading to a more potent induction of sterile protection.

In summary, we demonstrate that (1) the parasite liver load is not decisive for low levels of protection after ID-I compared to IV immunization, that (2) different types of hepatic immune responses are induced by IV- versus ID-I, with a preferred induction of regulatory hepatic T and B cells and a suboptimal CD8 memory T cell responses after ID immunization, and that (3) also in the skin-draining lymph node ID-I favors upregulation of regulatory immune responses. Understanding the suppressive immune responses after skin administration of sporozoites will be essential to improve ID immunization with attenuated sporozoites. Targeted inhibition of regulatory T and B cell function or prevention of their development may allow for the induction of more potent protective T cell responses by ID administered sporozoites and should help in development of skin-based vaccination strategies.

## Materials and Methods

### Experimental animals and wild type and transgenic P. yoelii lines

Female BALB/cByJ mice (6–7 weeks; Charles River, NL and Harlan, Bicester, UK) were used. All animal experiments of this study were approved by the Animal Experiments Committee of the Leiden University Medical Center (DEC 13132 and 14307). The Dutch Experiments on Animal Act is established under European guidelines (EU directive no. 86/609/EEC regarding the Protection of Animals used for Experimental and Other Scientific Purposes). All experiments were performed in accordance with relevant guidelines and regulations. Two *P. yoelii* (*Py*) lines were used: i) the reference ‘wild type’ *Py*17XNL parasite line 1971cl1 (Py-GFP-Luc_con_; line RMgm-689; www.pberghei.eu) which contains the fusion gene *gfp-luc* gene under control of the constitutive *eef1α* promoter integrated into the silent *230p* gene locus (PY17X_0306600) and does not contain a drug-selectable marker and ii) a *Py*17XNXL mutant that lacks the gene *fabb/f* (3-oxoacyl-acyl-carrier protein synthase; PY17X_1126500). This mutant (ΔPyFabBF-GFP-Luc_con_; mutant RMgm-4109; www.pberghei.eu) was generated in the reference line 1971cl1 (see above) by standard methods of transfection^98^ using a DNA construct that targets the *fabb/f* gene by double cross-over integration. The 5′- and 3′- *fabb/f* target regions were amplified using primers 7358 5′-at**gggccc**TTGCGCTATTTATAAGAGTTTGAGAGG/7359 5′-a**aggcct**CAAGAATATTTTTAAGGGCCATTTC and 7360 5′-ccgg**ggtacc**CAATGATTGCAAATACACCATCAG/7361 5′-ataagaatg**cggccgc**GTGGATATACGCAAGTGTGCGAG and cloned up (ApaI/StuI) and downstream (KpnI/NotI) of the *hdhfr/fcu* selectable marker cassette of plasmid pL0034 (MRA-849, www.beiresources.org). The final DNA construct was linearized with HindIII/EcoRI before transfection. Transfection, selection and cloning of transformed parasites was performed using standard genetic modification technologies^98^ using *Py* 1971cl1 as the parent parasite line. Cloned parasite lines were obtained (exp. 2251; ΔPyFabBF-GFP-Luc_con_) by the method of limiting dilution. Correct integration of DNA construct and disruption of gene was verified by Southern analyses of Pulsed Field Gel (PFG)-separated chromosomes and PCR analysis^98^. PFG-separated chromosomes were hybridized with a mixed probe of the human *dhfr* gene^99^ and ~800 bp fragment of 5′UTR of PBANKA_0508000 located on chromosome 5^100^. Primers used to confirm by PCR correct integration of the construct are listed in Fig. S1.

### Mosquito infection, preparation and injection of sporozoites

Sporozoites were obtained by manual dissection of the salivary glands of infected female *Anopheles stephensi* mosquitoes 14 days after feeding on infected mice. Mosquitoes were kept at a temperature of 24.5 °C and 80% humidity. Salivary glands were collected in RPMI medium, homogenized and filtered (40 µm Falcon, Corning, Amsterdam, NL). The free sporozoites were counted in a Bürker counting chamber using phase-contrast microscopy. For IV administration sporozoites were suspended in RPMI medium and per mouse 200 μl was injected into the tail vein. For ID administration sporozoites were also suspended in RPMI medium and per mouse 20 µl was injected (10 µl at each upper thigh). For ID injection 30 G × 8 mm needles (BD Micro-Fine, BD Biosciences, Breda, NL) were used and prior to administration of sporozoites, mice were anesthetized using isoflurane and shaved to optimize ID administration.

### Immunization protocol and prepatent period after challenge

For the immunization-challenge experiments mice were immunized with a primary immunization on day 0 and a boost immunization on day 14 using isolated ΔPyFabBF-GFP-Luc_con_ sporozoites. For IV immunization 10 K sporozoites were used and for IV immunization 50 K ID. Blood of immunized mice was analyzed for possible breakthrough blood infections by Giemsa-stained blood smears after each immunization. Immunized mice and naïve controls were challenged 14 days after the last immunization with 10 K wt Py-GFP-Luc_con_ sporozoites or by bite of 10 infected mosquitoes. Challenged mice were monitored for blood-stage infections by Giemsa-stained blood smear during days 4–14 after challenge. The prepatent period (measured in days after sporozoite challenge) is defined as the day when a blood stage infection with a parasitemia of 0.5–2% is observed. Organs used for immunological analysis were collected 7 days after sporozoite immunization or 7 days after challenge.

### Artesunate treatment of challenged mice

Artesunate powder (Sigma-Aldrich, Zwijndrecht, NL) was dissolved in 5% sodium bicarbonate (NaHCO_3_) in drinking water of the mice. Based on a daily intake of mice of 4–7 ml drinking water per day^15^, we used a concentration of 0.3 mg/ml artesunate in the drinking water resulting in a daily oral dose of 1.2 to 2.1 mg (60 mg/kg bodyweight). This dose has been shown to effectively kill blood stage parasites in rodent malaria^46^. The drug in the drinking water was given to mice at the day of challenge until organ dissection for analysis of immune responses.

### Determination of parasite liver load after immunization and challenge by real time *in vivo* imaging

Parasite liver loads in live mice were quantified by real time *in vivo* imaging as previously described^101, 102^. Liver stages were visualized and liver loads quantified by measuring luciferase activity of parasites in whole bodies of mice at 44 h after injection of sporozoites using the IVIS Lumina II Imaging System (Perkin Elmer Life Sciences, Waltham, USA). Before measurements the fur from bellies of the mice were shaved and during measurements mice were anesthetized using the isofluorane-anesthesia system (XGI-8, Caliper Life Sciences, Hopkinton, USA). D-luciferin was dissolved in PBS (100 mg/kg; Caliper Life Sciences, USA) and injected subcutaneously in the neck. Measurements were performed within 8 minutes after the injection of D-luciferin. Quantitative analysis of bioluminescence of whole bodies was performed by measuring the luminescence signal intensity using the ROI (region of interest) settings of the Living Image® 4.4 software.

### Liver perfusion and cell purification

Mice were perfused under anaesthesia by intracardiac injection of 20 ml phosphate buffered saline (PBS, B. Braun, Oss, NL). Perfused livers were minced in small pieces and digested for 45 min at 37 °C in HBSS (Thermo Fisher Scientific, Breda, NL) containing 1 mg/ml type IV collagenase from *Clostridium histolyticum*, 2000 U/ml DNase 1 (both Sigma-Aldrich, NL) and 1 mM CaCl_2_. Single-cell suspension was obtained by passing the digested tissue through a 100 µM cell-strainer, and hepatocytes removed by centrifugation at low speed (50x g, 3 min). The supernatant containing hepatic leukocytes was centrifuged at 1500 rpm, 10 min, and red blood cells lyzed in the cell pellet by 2 min incubation with cold lysis buffer (0.15 M NH_4_Cl, 1 mM KHCO_3_, 0.1 mM Na_2_EDTA in PBS). PBS supplemented with 1% heat-inactivated fetal calf serum (FCS; Greiner Bio-One, Alphen aan den Rijn, NL) and 2.5 mM EDTA (Sigma-Aldrich) was used for rinsing and washing steps. Leukocytes were isolated by positive selection using anti-CD45 Microbeads (Miltenyi Biotec, Leiden, NL; 35 µl beads per liver). Purity was generally >95%.

### Blood collection

Blood (~500–700 μl) was collected from the orbital sinus of anaesthetized mice into heparin-coated tubes. After red blood cell lysis by 5 min incubation with cold lysis buffer, PBMC were obtained for subsequent *in vitro* culture or flow cytometric analysis.

### Collection of lymph node cells

Inguinal lymph nodes were digested for 20 min in RPMI (1640 glutamax; Thermo Fisher Scientific) containing Collagenase D (1 mg/ml; Roche Life Science, Almere, The Netherlands) and DNase 1 (400 U/mL; Sigma-Aldrich) at 37 °C. Single-cell suspension was obtained by passing the digested tissue through a 100 µM cell-strainer.

### *In vitro* cultures of hepatic leukocytes, PBMC or lymph node cells

For detection of intracellular cytokines and cytotoxicity-related proteins, hepatic leukocytes, PBMC or lymph node cells (2.5 × 10^6^/ml) were cultured in medium (RPMI 1640 glutamax; Thermo Fisher Scientific), containing 5% FCS, 5 × 10^−5^ M 2-Mercaptoethanol (Sigma-Aldrich) and antibiotics (100 U/ml penicillin and 100 μg/ml streptomycin; both Sigma-Aldrich). *P. yoelii* circumsporozoite peptide (CSP; SYVPSAEQI^103^) (10 µg/ml) was added for 4 h for flow cytometric detection of IFN-γ, TNF, IL-12p40, granzyme B and CD107a. Either brefeldin A (10 µg/ml) or a combination of brefeldin A, phorbol myristate acetate (PMA, 100 ng/ml) and ionomycin (1 μg/ml) (all Sigma-Aldrich) was added to the cells as indicated in the figure legends. For the detection of intracellular IL-10 it was required to culture cells with CSP peptide (10 µg/ml) plus *P. yoelii* sporozoites (*Py* wt-GFP-Luc_con_, 25,000/ml) for 36 h including PMA, ionomycin and brefeldin A in the last 4 h. For flow cytometric detection of surface-exposed CD107a, cells were cultured for 4 hours with 0.25% GolgiStop (BD Biosciences), 10 µg/ml brefeldin A and anti-mouse CD107a-AlexaFluor488 (eBioscience, Vienna, Austria). For the detection of secreted IFN-γ, cells were cultured with CSP peptide and *P. yoelii* sporozoites for 36 h prior to collection of supernatants.

### Flow cytometry

Cells were stained with the live/dead marker Aqua (Thermo Fisher Scientific) and fixed with the eBioscience Foxp3/Transcription Factor kit or with 1.9% paraformaldehyde (Sigma-Aldrich). For the surface staining of the degranulation marker CD107a, cells were left unfixed and directly processed for flow cytometric analysis. For intracellular staining, cells were permeabilized with the eBioscience kit or 0.5% saponin (Sigma-Aldrich). Flow cytometric analysis was performed by staining with fluorochrome-labeled antibodies against mouse CD3, CD19, CD44, CD45RB, CD107a, CTLA-4, F4/80, Foxp3, GzmB, IL-10, IL-12p40, MHCII, TNF (all eBioscience), CD11c, CD25, IFN-γ (BD Biosciences), and CD8, CD64, GITR, PD-1 and PD-ligand 1 (Biolegend, Uithoorn, NL). For all flow cytometric stainings, FcγR-binding inhibitor (2.4G2) was added and fluorescence minus one (FMO) controls were used for gate setting. Flow cytometry was performed using a FACSCanto (BD Biosciences).

### ELISA

After 36 h *in vitro* culture of hepatic leukocytes or PBMC with CSP peptide and *P. yoelii* sporozoites as described above, the concentration of IFN-γ in the culture supernatant was determined by a commercial ELISA kit according to the manufacturer’s instructions (BD Biosciences). Levels of CSP-antibodies in mice were determined by ELISA. Mouse sera of naïve mice were used as negative control. The 96-well ELISA plates were first coated with 10 K of lyzed wt Py-GFP-Luc_con_ sporozoites overnight (pellet resuspended in NaHCO_3_ buffer (pH 9,6)) overnight and then blocked with 1% BSA in PBS-Tween. Mouse sera were diluted (1/10 000) and added to the plates and incubated for 3 h. After washing the plates, HRP-conjugated rabbit anti-mouse IgG Fc Fragment was added (1/5000; incubation for 1 h). After TMB High Sensitivity Substrate was added, ODs were read at 450 nm.

### Statistics

Statistical analyses were performed using unpaired t-test or Mann-Whitney test with the GraphPad Prism software package 6.05 (GraphPad Software, Inc). One-sample t-test of log-transformed data was applied to calculate significant changes for data which are expressed as fold increase. Correlation coefficients were obtained by Spearman correlation analysis. *p*-values < 0.05 were considered significant. All data are presented as mean ± standard error of the mean (SEM).

## Electronic supplementary material

Supplementary Information

## References

[1] Luke TC, Hoffman SL (2003). Rationale and plans for developing a non-replicating, metabolically active, radiation-attenuated *Plasmodium falciparum* sporozoite vaccine. The Journal of experimental biology.

[2] Moorthy VS, Newman RD, Okwo-Bele JM (2013). Malaria vaccine technology roadmap. Lancet.

[3] Pinzon-Charry A, Good MF (2008). Malaria vaccines: the case for a whole-organism approach. Expert opinion on biological therapy.

[4] Hoffman SL, Vekemans J, Richie TL, Duffy PE (2015). The March Toward Malaria Vaccines. American journal of preventive medicine.

[5] Seder RA (2013). Protection against malaria by intravenous immunization with a nonreplicating sporozoite vaccine. Science.

[6] Hoffman SL (2002). Protection of humans against malaria by immunization with radiation-attenuated *Plasmodium falciparum* sporozoites. The Journal of infectious diseases.

[7] Roestenberg M (2011). Long-term protection against malaria after experimental sporozoite inoculation: an open-label follow-up study. Lancet.

[8] Roestenberg M (2009). Protection against a malaria challenge by sporozoite inoculation. The New England journal of medicine.

[9] Nussenzweig RS, Vanderberg J, Most H, Orton C (1967). Protective immunity produced by the injection of x-irradiated sporozoites of *Plasmodium berghei*. Nature.

[10] Nussenzweig RS, Vanderberg JP, Most H, Orton C (1969). Specificity of protective immunity produced by x-irradiated *Plasmodium berghei* sporozoites. Nature.

[11] Hoffman SL (2010). Development of a metabolically active, non-replicating sporozoite vaccine to prevent *Plasmodium falciparum* malaria. Human vaccines.

[12] Ploemen IH (2013). *Plasmodium* liver load following parenteral sporozoite administration in rodents. Vaccine.

[13] Nganou-Makamdop K (2012). Reduced *Plasmodium berghei* sporozoite liver load associates with low protective efficacy after intradermal immunization. Parasite immunology.

[14] Parmar R (2016). Route of administration of attenuated sporozoites is instrumental in rendering immunity against *Plasmodia* infection. Vaccine.

[15] Mueller AK (2005). *Plasmodium* liver stage developmental arrest by depletion of a protein at the parasite-host interface. Proceedings of the National Academy of Sciences of the United States of America.

[16] Voza T, Kebaier C, Vanderberg JP (2010). Intradermal immunization of mice with radiation-attenuated sporozoites of *Plasmodium yoelii* induces effective protective immunity. Malaria journal.

[17] Inoue M, Culleton RL (2011). The intradermal route for inoculation of sporozoites of rodent malaria parasites for immunological studies. Parasite immunology.

[18] Mac-Daniel L (2014). Local immune response to injection of *Plasmodium* sporozoites into the skin. Journal of immunology (Baltimore, Md.: 1950).

[19] Hickling JK (2011). Intradermal delivery of vaccines: potential benefits and current challenges. Bulletin of the World Health Organization.

[20] Douradinha B (2007). Genetically attenuated P36p-deficient *Plasmodium berghei* sporozoites confer long-lasting and partial cross-species protection. International journal for parasitology.

[21] Epstein JE (2011). Live attenuated malaria vaccine designed to protect through hepatic CD8(+) T cell immunity. Science.

[22] Bastiaens GJ (2016). Safety, Immunogenicity, and protective efficacy of intradermal immunization with aseptic, purified, cryopreserved *Plasmodium falciparum* sporozoites in volunteers under chloroquine prophylaxis: a randomized controlled trial. The American journal of tropical medicine and hygiene.

[23] Guilbride DL, Gawlinski P, Guilbride PD (2010). Why functional pre-erythrocytic and bloodstage malaria vaccines fail: a meta-analysis of fully protective immunizations and novel immunological model. PLoS One.

[24] Pfeil J, Heine JF, Mueller AK (2015). Addition of histamine to subcutaneously injected *Plasmodium berghei* sporozoites increases the parasite liver load and could facilitate whole-parasite vaccination. Malaria journal.

[25] Chatterjee S, Druilhe P, Wery M (1999). Irradiated sporozoites prime mice to produce high antibody titres upon viable *Plasmodium berghei* sporozoite challenge, which act upon liver-stage development. Parasitology.

[26] Mellouk S, Lunel F, Sedegah M, Beaudoin RL, Druilhe P (1990). Protection against malaria induced by irradiated sporozoites. Lancet.

[27] Mueller AK (2007). Genetically attenuated *Plasmodium berghei* liver stages persist and elicit sterile protection primarily via CD8 T cells. The American journal of pathology.

[28] Scheller LF, Azad AF (1995). Maintenance of protective immunity against malaria by persistent hepatic parasites derived from irradiated sporozoites. Proceedings of the National Academy of Sciences of the United States of America.

[29] Silvie O (2002). Effects of irradiation on *Plasmodium falciparum* sporozoite hepatic development: implications for the design of pre-erythrocytic malaria vaccines. Parasite immunology.

[30] van Dijk MR (2005). Genetically attenuated, P36p-deficient malarial sporozoites induce protective immunity and apoptosis of infected liver cells. Proceedings of the National Academy of Sciences of the United States of America.

[31] Vanderberg JP, Nussenzweig RS, Most H, Orton CG (1968). Protective immunity produced by the injection of x-irradiated sporozoites of *Plasmodium berghei*. II. Effects of radiation on sporozoites. The Journal of parasitology.

[32] Berenzon D (2003). Protracted protection to *Plasmodium berghei* malaria is linked to functionally and phenotypically heterogeneous liver memory CD8+ T cells. Journal of immunology (Baltimore, Md.: 1950).

[33] Guebre-Xabier M, Schwenk R, Krzych U (1999). Memory phenotype CD8(+) T cells persist in livers of mice protected against malaria by immunization with attenuated *Plasmodium berghei* sporozoites. European journal of immunology.

[34] Jobe O (2009). Immunization with radiation-attenuated *Plasmodium berghei* sporozoites induces liver cCD8alpha + DC that activate CD8+ T cells against liver-stage malaria. PLoS One.

[35] Jobe O (2007). Genetically attenuated *Plasmodium berghei* liver stages induce sterile protracted protection that is mediated by major histocompatibility complex Class I-dependent interferon-gamma-producing CD8+ T cells. The Journal of infectious diseases.

[36] Ponnudurai T, Lensen AH, van Gemert GJ, Bolmer MG, Meuwissen JH (1991). Feeding behaviour and sporozoite ejection by infected *Anopheles stephensi*. Transactions of the Royal Society of Tropical Medicine and Hygiene.

[37] Vaughan JA, Scheller LF, Wirtz RA, Azad AF (1999). Infectivity of *Plasmodium berghei* sporozoites delivered by intravenous inoculation versus mosquito bite: implications for sporozoite vaccine trials. Infection and immunity.

[38] Lin JW (2011). A novel ‘gene insertion/marker out’ (GIMO) method for transgene expression and gene complementation in rodent malaria parasites. PLoS One.

[39] Annoura T (2014). Two *Plasmodium* 6-Cys family-related proteins have distinct and critical roles in liver-stage development. FASEB J.

[40] Dankwa DA, Davis MJ, Kappe SH, Vaughan AM (2016). *A Plasmodium yoelii* Mei2-Like RNA Binding Protein Is Essential for Completion of Liver Stage Schizogony. Infection and immunity.

[41] Vaughan AM (2009). Type II fatty acid synthesis is essential only for malaria parasite late liver stage development. Cellular microbiology.

[42] Annoura T, Chevalley S, Janse CJ, Franke-Fayard B, Khan SM (2013). Quantitative analysis of *Plasmodium berghei* liver stages by bioluminescence imaging. Methods in molecular biology (Clifton, N.J.).

[43] Ocana-Morgner C, Mota MM, Rodriguez A (2003). Malaria blood stage suppression of liver stage immunity by dendritic cells. The Journal of experimental medicine.

[44] Medeiros MM (2013). Liver accumulation of *Plasmodium chabaudi*-infected red blood cells and modulation of regulatory T cell and dendritic cell responses. PLoS One.

[45] Bijker EM (2015). Studying the effect of chloroquine on sporozoite-induced protection and immune responses in *Plasmodium berghei* malaria. Malaria journal.

[46] Peng X (2014). Artesunate versus chloroquine infection-treatment-vaccination defines stage-specific immune responses associated with prolonged sterile protection against both pre-erythrocytic and erythrocytic *Plasmodium yoelii* infection. Journal of immunology (Baltimore, Md.: 1950).

[47] Radtke AJ, Tse SW, Zavala F (2015). From the draining lymph node to the liver: the induction and effector mechanisms of malaria-specific CD8+ T cells. Seminars in immunopathology.

[48] Krzych U, Zarling S, Pichugin A (2014). Memory T cells maintain protracted protection against malaria. Immunology letters.

[49] Hafalla JC, Cockburn IA, Zavala F (2006). Protective and pathogenic roles of CD8+ T cells during malaria infection. Parasite immunology.

[50] Tsuji M, Zavala F (2003). T cells as mediators of protective immunity against liver stages of *Plasmodium*. Trends Parasitology.

[51] Schmidt NW, Butler NS, Badovinac VP, Harty JT (2010). Extreme CD8 T cell requirements for anti-malarial liver-stage immunity following immunization with radiation attenuated sporozoites. PLoS Pathogens.

[52] Schwenk R (2014). IgG2 antibodies against a clinical grade *Plasmodium falciparum* CSP vaccine antigen associate with protection against transgenic sporozoite challenge in mice. PLoS One.

[53] Foquet L (2014). Vaccine-induced monoclonal antibodies targeting circumsporozoite protein prevent *Plasmodium falciparum* infection. The Journal of clinical investigation.

[54] Sakaguchi S, Wing K, Onishi Y, Prieto-Martin P, Yamaguchi T (2009). Regulatory T cells: how do they suppress immune responses?. International immunology.

[55] Boer MC, Joosten SA, Ottenhoff TH (2015). Regulatory T-Cells at the Interface between Human Host and Pathogens in Infectious Diseases and Vaccination. Frontiers in immunology.

[56] Roncarolo MG, Gregori S, Bacchetta R, Battaglia M (2014). Tr1 cells and the counter-regulation of immunity: natural mechanisms and therapeutic applications. Current topics in microbiology and immunology.

[57] Zarling S, Krzych U (2015). Characterization of liver CD8 T cell subsets that are associated with protection against pre-erythrocytic *Plasmodium* parasites. Methods in molecular biology (Clifton, N.J.).

[58] Van Braeckel-Budimir N, Harty JT (2014). CD8 T-cell-mediated protection against liver-stage malaria: lessons from a mouse model. Frontiers in microbiology.

[59] Nganou-Makamdop K, van Gemert GJ, Arens T, Hermsen CC, Sauerwein RW (2012). Long term protection after immunization with *P. berghei* sporozoites correlates with sustained IFNgamma responses of hepatic CD8+ memory T cells. PLoS One.

[60] Wykes MN, Horne-Debets JM, Leow CY, Karunarathne DS (2014). Malaria drives T cells to exhaustion. Frontiers in microbiology.

[61] Montagna, G. N., Biswas, A., Hildner, K., Matuschewski, K., Dunay, I. R. Batf3 deficiency proves the pivotal role of CD8alpha + dendritic cells in protection induced by vaccination with attenuated *Plasmodium* sporozoites. *Parasite immunology* (2015). 10.1111/pim.12222 26284735

[62] Spencer, A. J. *et al*. The threshold of protection from liver-stage malaria relies on a fine balance between the number of infected hepatocytes and effector CD8+ T Cells present in the liver. Journal of immunology (Baltimore, Md.: 1950) (2017). 10.4049/jimmunol.1601209 PMC5318841 28087668

[63] Olotu A (2011). Circumsporozoite-specific T cell responses in children vaccinated with RTS,S/AS01E and protection against *P. falciparum* clinical malaria. PLoS One.

[64] Leiriao P, Mota MM, Rodriguez A (2005). Apoptotic *Plasmodium*-infected hepatocytes provide antigens to liver dendritic cells. The Journal of infectious diseases.

[65] Chakravarty S (2007). CD8+ T lymphocytes protective against malaria liver stages are primed in skin-draining lymph nodes. Nature medicine.

[66] Wakim LM, Waithman J, van Rooijen N, Heath WR, Carbone FR (2008). Dendritic cell-induced memory T cell activation in nonlymphoid tissues. Science.

[67] Leon, B., Lopez-Bravo, M. & Ardavin, C. Monocyte-derived dendritic cells formed at the infection site control the induction of protective T helper 1 responses against *Leishmania*. *Immunity* **26**, 519–531 (2007). 10.1016/j.immuni.2007.01.017 17412618

[68] Qu C, Nguyen VA, Merad M, Randolph GJ (2009). MHC class I/peptide transfer between dendritic cells overcomes poor cross-presentation by monocyte-derived APCs that engulf dying cells. Journal of immunology (Baltimore, Md.: 1950).

[69] Cheong C (2010). Microbial stimulation fully differentiates monocytes to DC-SIGN/CD209(+) dendritic cells for immune T cell areas. Cell.

[70] Qu C, Brinck-Jensen NS, Zang M, Chen K (2014). Monocyte-derived dendritic cells: targets as potent antigen-presenting cells for the design of vaccines against infectious diseases. International journal of infectious diseases: IJID: official publication of the International Society for Infectious Diseases.

[71] Couper KN (2008). IL-10 from CD4CD25Foxp3CD127 adaptive regulatory T cells modulates parasite clearance and pathology during malaria infection. PLoS Pathogens.

[72] Linke A (1996). *Plasmodium chabaudi chabaudi*: differential susceptibility of gene-targeted mice deficient in IL-10 to an erythrocytic-stage infection. Experimental Parasitology.

[73] Espinoza Mora MR (2014). Depletion of regulatory T cells augments a vaccine-induced T effector cell response against the liver-stage of malaria but fails to increase memory. PLoS One.

[74] Van Braeckel-Budimir N, Kurup SP, Harty JT (2016). Regulatory issues in immunity to liver and blood-stage malaria. Current opinion in immunology.

[75] Chen G (2009). Effects of CD4(+)CD25(+)Foxp3(+)regulatory T cells on early *Plasmodium yoelii* 17XL infection in BALB/c mice. Parasitology.

[76] Lyke KE (2004). Serum levels of the proinflammatory cytokines interleukin-1 beta (IL-1beta), IL-6, IL-8, IL-10, tumor necrosis factor alpha, and IL-12(p70) in Malian children with severe *Plasmodium falciparum* malaria and matched uncomplicated malaria or healthy controls. Infection and immunity.

[77] Dodoo D (2002). Absolute levels and ratios of proinflammatory and anti-inflammatory cytokine production *in vitro* predict clinical immunity to *Plasmodium falciparum* malaria. The Journal of infectious diseases.

[78] Ho M (1998). Endogenous interleukin-10 modulates proinflammatory response in *Plasmodium falciparum* malaria. The Journal of infectious diseases.

[79] Majlessi L, Lo-Man R, Leclerc C (2008). Regulatory B and T cells in infections. Microbes and infection.

[80] Ronet C (2010). Regulatory B cells shape the development of Th2 immune responses in BALB/c mice infected with *Leishmania* major through IL-10 production. Journal of immunology (Baltimore, Md.: 1950).

[81] Jeong YI (2014). Identification of anti-allergic effect of *Clonorchis sinensis*-derived protein venom allergen-like proteins (CsVAL). Biochemical and biophysical research communications.

[82] Hussaarts L, van der Vlugt LE, Yazdanbakhsh M, Smits HH (2011). Regulatory B-cell induction by helminths: implications for allergic disease. The Journal of allergy and clinical immunology.

[83] Wilson MS (2010). Helminth-induced CD19 + CD23hi B cells modulate experimental allergic and autoimmune inflammation. European journal of immunology.

[84] Liu Y (2013). Role of IL-10-producing regulatory B cells in control of cerebral malaria in *Plasmodium berghei* infected mice. European journal of immunology.

[85] Foulds KE, Rotte MJ, Seder RA (2006). IL-10 is required for optimal CD8 T cell memory following *Listeria monocytogenes* infection. Journal of immunology (Baltimore, Md.: 1950).

[86] Laidlaw BJ (2015). Production of IL-10 by CD4(+) regulatory T cells during the resolution of infection promotes the maturation of memory CD8(+) T cells. Nature immunology.

[87] Tian Y, Mollo SB, Harrington LE, Zajac AJ (2016). IL-10 regulates memory T cell development and the balance between Th1 and follicular Th cell responses during an acute viral infection. Journal of immunology (Baltimore, Md.: 1950).

[88] Belkaid Y, Piccirillo CA, Mendez S, Shevach EM, Sacks DL (2002). CD4+ CD25+ regulatory T cells control *Leishmania major* persistence and immunity. Nature.

[89] Shin SC, Vanderberg JP, Terzakis JA (1982). Direct infection of hepatocytes by sporozoites of *Plasmodium berghei*. The Journal of protozoology.

[90] Yamauchi LM, Coppi A, Snounou G, Sinnis P (2007). *Plasmodium* sporozoites trickle out of the injection site. Cellular microbiology.

[91] Apte SH (2013). Subcutaneous cholera toxin exposure induces potent CD103(+) dermal dendritic cell activation and migration. European journal of immunology.

[92] Stein P (2011). Regulatory T cells and IL-10 independently counterregulate cytotoxic T lymphocyte responses induced by transcutaneous immunization. PLoS One.

[93] Guilbride DL, Guilbride PD, Gawlinski P (2012). Malaria’s deadly secret: a skin stage. Trends in Parasitology.

[94] Bijker EM, Schats R, Visser LG, Sauerwein RW, Scholzen A (2015). *Ex vivo* lymphocyte phenotyping during *Plasmodium falciparum* sporozoite immunization in humans. Parasite immunology.

[95] Bijker EM (2014). Cytotoxic markers associate with protection against malaria in human volunteers immunized with *Plasmodium falciparum* sporozoites. The Journal of infectious diseases.

[96] Eng NF, Bhardwaj N, Mulligan R, Diaz-Mitoma F (2013). The potential of 1018 ISS adjuvant in hepatitis B vaccines: HEPLISAV review. Human vaccines & immunotherapeutics.

[97] Hung IF (2016). Topical imiquimod before intradermal trivalent influenza vaccine for protection against heterologous non-vaccine and antigenically drifted viruses: a single-centre, double-blind, randomised, controlled phase 2b/3 trial. The Lancet. Infectious diseases.

[98] Janse CJ, Ramesar J, Waters AP (2006). High-efficiency transfection and drug selection of genetically transformed blood stages of the rodent malaria parasite *Plasmodium berghei*. Nature Protocols.

[99] Braks JA, Franke-Fayard B, Kroeze H, Janse CJ, Waters AP (2006). Development and application of a positive-negative selectable marker system for use in reverse genetics in *Plasmodium*. Nucleic Acids Research.

[100] Fougere A (2016). Variant exported blood-stage proteins encoded by *Plasmodium* multigene families are expressed in liver stages where they are exported into the parasitophorous vacuole. PLoS Pathog.

[101] Rijpma SR (2016). Vital and dispensable roles of *Plasmodium* multidrug resistance transporters during blood- and mosquito-stage development. Molecular microbiology.

[102] van der Velden M (2016). Protective efficacy induced by genetically attenuated mid-to-late liver-stage arresting *Plasmodium berghei* Deltamrp2 parasites. The American journal of tropical medicine and hygiene.

[103] Sano G (2001). Swift development of protective effector functions in naive CD8(+) T cells against malaria liver stages. The Journal of experimental medicine.

