# Supplementary material for: Protective immunity differs between routes of administration of attenuated malaria parasites independent of parasite liver load
Source: Sci Rep. 2017 Sep 4;7:10372. doi: 10.1038/s41598-017-10480-1 (PMC5583236; doi:10.1038/s41598-017-10480-1)
Supplement: Supplementary file 1 — Supplementary Information [file 41598_2017_10480_MOESM1_ESM.pdf]

Protective immunity differs between routes of administration of attenuated malaria parasites independent of parasite liver load

Simone Haeberlein[1#], Séverine Chevalley-Maurel[1], Arifa Ozir-Fazalalikhan[1], Hester Koppejan[1], Beatrice M. F. Winkel[1], Jai Ramesar[1], Shahid M. Khan[1], Robert W. Sauerwein[3], Meta Roestenberg[1,2], Chris J. Janse[1], Hermelijn H. Smits[1] and Blandine Franke-Fayard[1*]

Supplementary Figures

Figure S1. Generation and genotyping of parasites lacking expression of Py *fabb/f* (ΔPyFabBF-GFP-Lucc$_{on}$; line 2251cl3)

(A) Schematic representation of the plasmid, the wild-type gene *fabb/f* locus before and after incorporation of the construct pL1980 used to generate the deletion mutant (exp 2251). The construct contains the *human dihydrofolate reductase* (*hdhfr*) / *fcu* selectable marker cassette (SM: white arrow) and two target regions of the *fabb/f* locus (back arrows) for integration by double cross-over homologous recombination. Primers and sizes of PCR products of panel C are indicated. (**B)** Diagnostic Southern and PCR analysis of pulsed field gel-separated chromosomes confirm correct integration of the *fabb/f* gene deletion in cloned line 2251cl3. Separated chromosomes were hybridized with *hdhfr* gene recognizing the SM cassette on chromosome 11 and a ~800bp fragment of chromosome 5. **(C)** Correct integration of the vector in *fabb/f* locus as shown by PCR. M: marker; SM: amplification of *hdhfr*; 5': verification of 5' integration fidelity; 3': verification of 3' integration fidelity and ORF: amplification of *fabb/f* gene. **(D)** Primer sequences.

Figure S2. Immunization and challenge protocol to study protection outcome and immune responses. BALB/c mice were immunized twice 2 weeks apart with liver-attenuated luciferase-expressing ΔPyFabBF-GFP-Luc$_{con}$ sporozoites (PyGAP) either with 10K intravenously (IV) or 50K intradermally (ID). Mice were either sacrificed to study hepatic immune responses at 7 days after

primary or boost injection, or were challenged IV with 10K luciferase-expressing wild-type 10K Py-GFP-Luc_{con} (PyWT) or by infected mosquito bites 2 weeks after the boost immunization. Challenged mice were monitored for blood-stage parasitemia by Giemsa-stained blood smear during days 4–14 after challenge, or during days 4-7 in case liver immunity was studied at day 7 after challenge. Parasite loads in liver were quantified at 44 h after each sporozoite administration by *in vivo* imaging of luciferase activity (IVIS).

Figure S3. Challenge by infected mosquito bites induces similar protection outcome as challenge by IV inoculation of sporozoites. Mice were immunized by IV or ID route and challenged by IV injection of 10K wt sporozoites (needle), or challenged by bites from 10 wt infected mosquitoes (mosquito). No artesunate treatment was applied to receive information about prepatency. **(A, B)** Luciferase activity at 44 h after challenge by mosquito bites. Naïve mice were challenged as a control. **(C)** Summary of protection outcome and days of prepatency of 5-8 mice per group. Significant difference by unpaired t-test is indicated by * p<0.05.

Figure S4. Immunization and challenge protocol to study immune responses under artesunate treatment. BALB/c mice were immunized at day 0 and 14 with liver-attenuated luciferase-expressing ΔPyFabBF-GFP-Luc_{con} sporozoites (PyGAP) either with 10K intravenously (IV) or 50K intradermally (ID). Mice were sacrificed to study hepatic immune responses at 7 days after IV challenge with 10K luciferase-expressing wild-type 10K Py-GFP-Luc_{con} (PyWT). To prevent blood stage parasitemia after wt challenge, immunized mice were orally treated with artesunate from the day of challenge infection until 7 days pi when immune responses of liver and PBMC were studied.

Figure S5. Comparable hepatic immune responses with and without artesunate treatment. IV immunized mice were treated with artesunate (art) starting at the day of challenge to prevent blood stage development, or left untreated (ctrl). Also naïve mice were treated with or without artesunate, but not challenged. Hepatic leukocytes were analyzed by flow cytometry at day 7 after challenge. **(A, B)** Frequency of $CD44^{hi}$ memory T cells within the CD8 (A) or CD4 (B) T cell population. **(C-E)** Intracellular IFN-γ expression of CD4 **(C)** or CD8 **(D, E)** T cells after 4 h culture of total leukocytes with brefeldin A plus CSP or PMA plus ionomycin (PI). **(F, G)** Intracellular IL-10 expression of $Foxp3^{+}CD25^{+}$ Treg cells **(F)** or $CD19^{+}$ B cells **(G)** after 36 h culture of leukocytes with CSP and sporozoites, and addition of brefeldin A and PI to the last 4 h. Summary of 4-7 mice per group. Significant difference by Mann-Whitney test is indicated by * p<0.05, ** p<0.01, *** p<0.001 (vs. respective naïve untreated or artesunate treated group), and $^{\#}$ p<0.05 (between immunized mouse groups).

Figure S6. Effector responses that were mostly similar between protected and unprotected ID immunized mice. Challenge was performed under artesunate treatment and hepatic leukocytes analyzed 7 days later. **(A)** Mean fluorescence intensity (MFI) of granzyme B (GzmB) in CD8 T cells after 4 h culture of leukocytes with CSP and brefeldin A. **(B, C)** Intracellular expression of IFN-γ (A) and TNF (B) of CD8 and CD4 T cells after 4 h culture of total leukocytes with PMA plus ionomycin (PI) and brefeldin A. **(D)** Frequency of $CD44^{hi}$ memory T cells within the CD8 and CD4 T cell populations. Summary of 8-14 mice per group. The dotted line indicates the mean cytokine or expression level for protected IV immunized mice (N=9-16). Significant difference by Mann-Whitney test is indicated by * p<0.05, ** p<0.01, *** p<0.001 (to naïve control group), and $^{\#}$ p<0.05, $^{\#\#}$ p<0.01 (between immunized mouse groups).

Figure S7. Effector immune responses in PBMC after challenge of IV or ID immunized mice. Mice were immunized by ID route, challenged under artesunate treatment, and distinguished into protected (p) and unprotected (unp) mice. PBMC were analyzed 7 days after challenge. **(A)** IFN-γ concentration as measured by ELISA in culture supernatant after 36 h stimulation of total PBMC with CSP and sporozoites. **(B)** Intracellular IFN-γ expression of $CD44^{hi}$ gated CD8 T cells after 4 h culture with CSP and brefeldin A. **(C)** Surface expression of CD107a on $CD44^{hi}$ CD8 T cells or CD4 T cells after 4 h culture of PBMC with CSP, brefeldin A and monensin. Summary of 2 experiments with 8-14 mice. The dotted line indicates the mean cytokine or expression level for protected IV immunized mice (N=9-16). Significant difference by Mann-Whitney test is indicated by ** $p<0.01$, *** $p<0.001$ (to naïve control group), and $^{\#}p<0.05$, $^{\#\#}p<0.01$ (between immunized mouse groups).

Figure S8. CSP-antibody titers in sera of naïve and IV and ID immunized mice. Seven days after challenge with live wild type Py sporozoites, the sera were collected and the antibody titers were determined by ELISA. ns, not significant.

Figure S9. Regulatory responses which were similar between protected and unprotected ID immunized mice. Hepatic Treg cells and B cells of protected and unprotected ID immunized mice were analyzed at day 7 after challenge under artesunate treatment. **(A)** $Foxp3^{+}CD25^{+}$ Treg cell frequency within the CD4 T cell population. **(B)** Treg cell numbers per liver. **(C)** Intracellular IL-10 expression of $CD19^{+}$ B cells after 36 h culture of leukocytes with CSP and sporozoites, and addition of PMA/ionomycin and brefeldin A to the last 4 h. Summary of 8-14 mice per group. The dotted line indicates the mean cytokine or expression level for protected IV immunized mice (N=9-16). Significant difference by Mann-Whitney test is indicated by * $p<0.05$, ** $p<0.01$, *** $p<0.001$ (to naïve control group).

Figure S10. IV and ID route of immunization induce similar high T cell effector responses in liver. Livers of IV or ID immunized mice were extracted at day 7 after primary immunization or boost. Livers of naïve mice were analyzed as control. **(A, B)** Intracellular IFN-γ expression of CD8$^+$ gated T cells after 4 h restimulation of hepatic leukocytes with (A) PMA/ionomycin (PI) in the presence of brefeldin A, or **(B)** CSP. **(C)** Intracellular expression of granzyme B (GzmB) of CD8 T cells after 4 h culture with CSP and brefeldin A. The histogram shows the expression level from one representative mouse per group. Summary of 2 experiments **(A, B)** or 1 representative out of 2 similar experiments **(C)** with 8-10 mice per group. Significant difference by Mann-Whitney test is indicated by ** $p<0.01$, *** $p<0.001$ (to naïve control group), and $^{\#\#}$ $p<0.01$ (between immunized mouse groups).

Figure S11. Exhaustion of hepatic T cells is comparable during IV and ID immunization. Mice were immunized by IV or ID route and hepatic leukocytes analyzed 7 days after prime-boost or boost. **(A-C)** Mean fluorescence intensity (MFI) of PD-1 surface expression of CD8 T cells **(A)**, CD4 T cells **(B)**, and Foxp3$^+$CD25$^+$ Treg cells **(C)** expressed as fold increase versus a naïve control mouse group (set as 1). **(D)** MFI of the ligand PD-L1 on MHCII$^+$CD11c^{hi} cDC, expressed as fold increase versus naïve control mice. Histograms show the expression level of one representative mouse of each group. Graphs show a summary of 5 mice per group. * $p<0.05$, ** $p<0.01$ indicates significant difference to the naïve control group by one-sample t-test of log-transformed data, and $^{\#}$ $p<0.05$ between immunized mouse groups by Mann-Whitey test.

Figure S12. Regulatory marker expression of Foxp3-negative CD4 T cells after immunization. Hepatic leukocytes were analyzed 7 days after IV or ID immunization by flow cytometry for surface expression of regulatory markers. Surface expression of the suppressive markers CTLA-4 **(A)** and GITR **(B)** on CD4$^+$Foxp3$^-$ T cells. Summary of 2 experiments with 8-10 mice per group. **(C)** Representative FACS plots of intracellular IFN-γ and IL-10 expression in Foxp3-negative CD4 T cells after 36 h culture of hepatic leukocytes with CSP and sporozoites and **(D)** frequencies of IL-10$^+$IFN-γ^+

double positive and IL-10^{+}IFN-γ^{-} single positive Foxp3-negative CD4 T cells (summary of n=5 mice, after primary immunization). Significant difference by Mann-Whitney test is indicated by * $p<0.05$, ** $p<0.01$, *** $p<0.001$ (to naïve control group), and $^{\#}$ $p<0.05$ (between immunized mouse groups).

Supplementary Tables

Table S1: Cell number of different cell types per liver at day 7 after primary immunization or boost by IV or ID route of sporozoite administration.

Table S2: Frequency of different cell types per inguinal lymph node at day 7 after primary immunization by IV or ID route of sporozoite administration.

Figure S1. Generation and genotyping of parasites lacking expression of Py *fabb/f* (ΔPyFabBF-GFP-Luc$_{con}$; line 2251cl3)

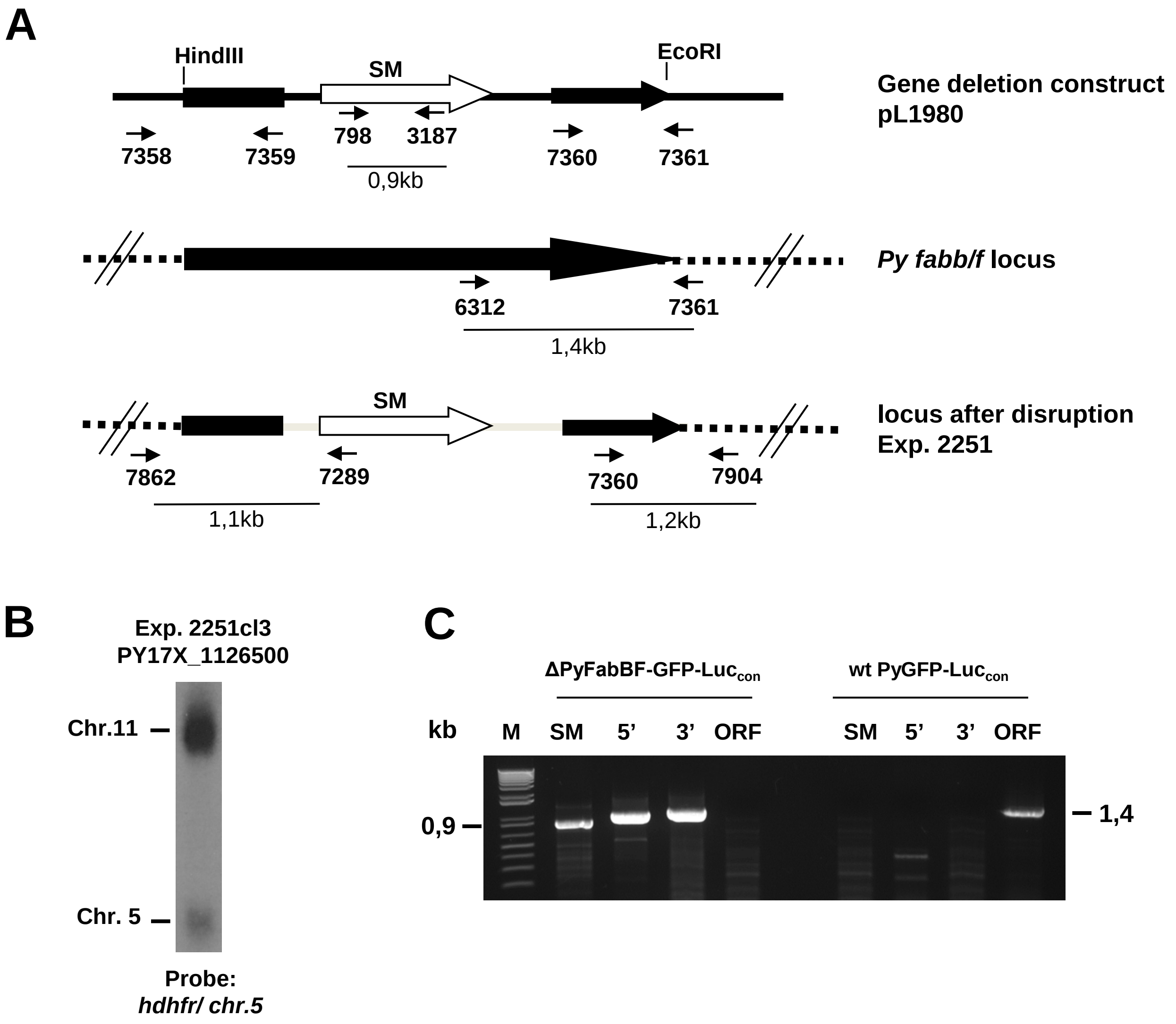

D

Primer	Location	Sequence
L798	SM-F	GCTCACCCCTCAAAGCACCAG
3187	SM-R	GTGTCACTTTCAAAGTCTTGC
7862	5'	CATTTGTTGCATATTTAGGTAATG
7289	5'	TAAAGCACAATATCTAGGATACTAC
7360	3'-F	CCGGGGTACCCAATGATTGCAAATACACCATCAG
7904	3'-R	GTTGCAATTTCCCCTACAAC
6312	ORF-F	GACTTCCAGAGTTGTATGCAC
7361	ORF-R	ATAAGAATGCGGCCGCGTGGATATACGCAAGTGTGCGAG

Figure S2. Immunization and challenge protocol to study protection outcome and immune responses

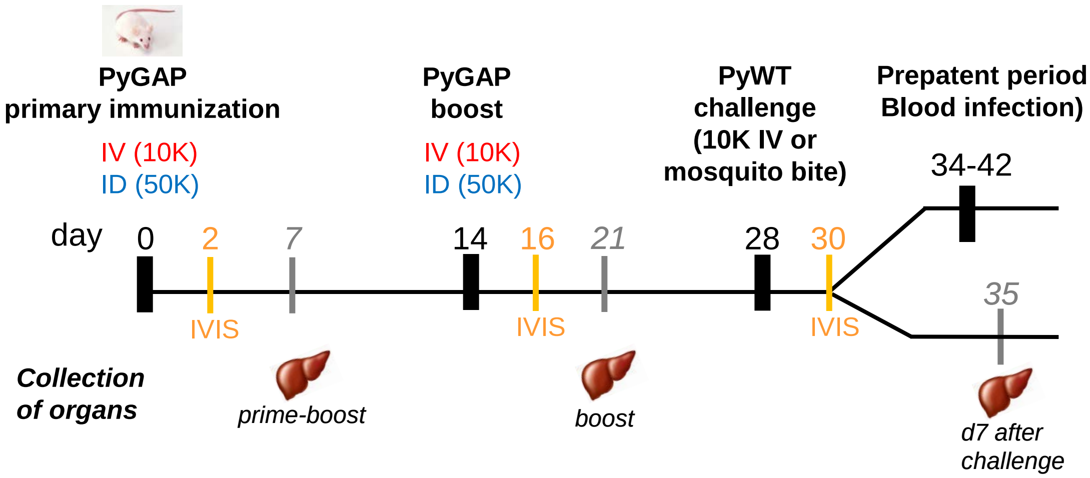

Figure S3: Challenge by infected mosquito bites induces similar protection outcome as challenge by IV inoculation of sporozoites

A

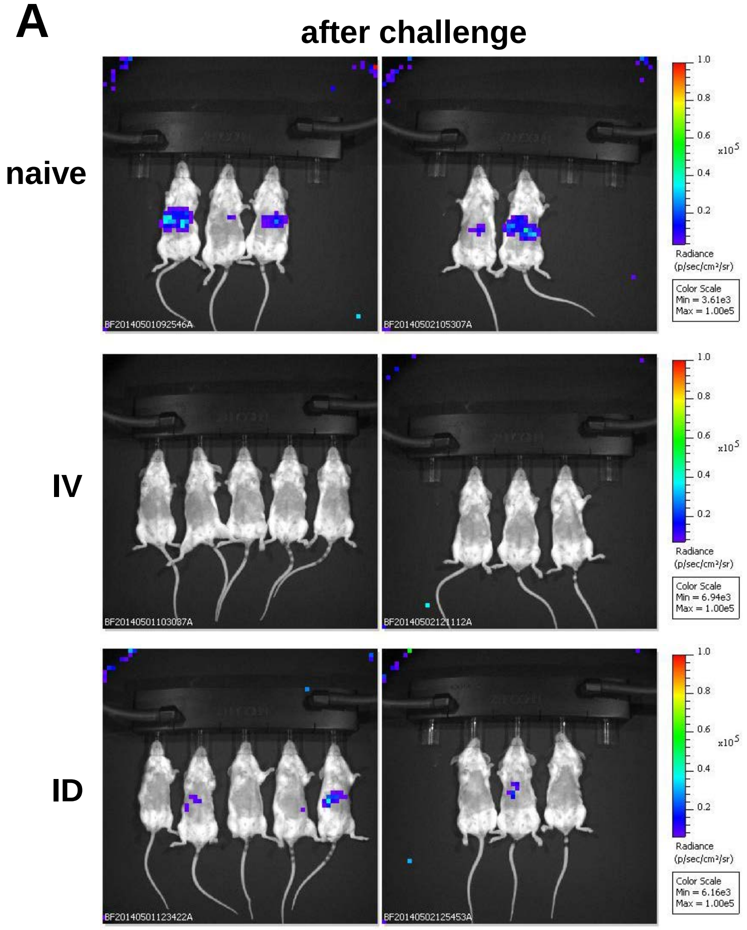

B

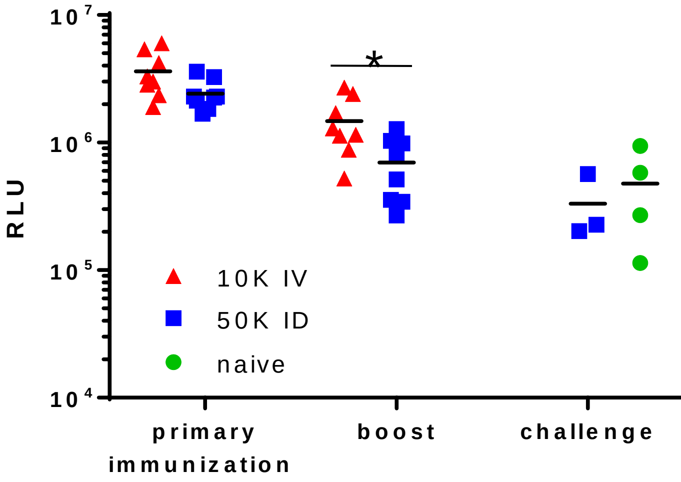

C

Group	No. of mice	No. unpro-tected	Protec-tion level	Prepa-tent period (days)
naive	5	5	0%	5-7
10K IV	8	1	88%	7
50K ID	8	3	63%	6-7

Figure S4. Immunization and challenge protocol to study immune responses under artesunate treatment

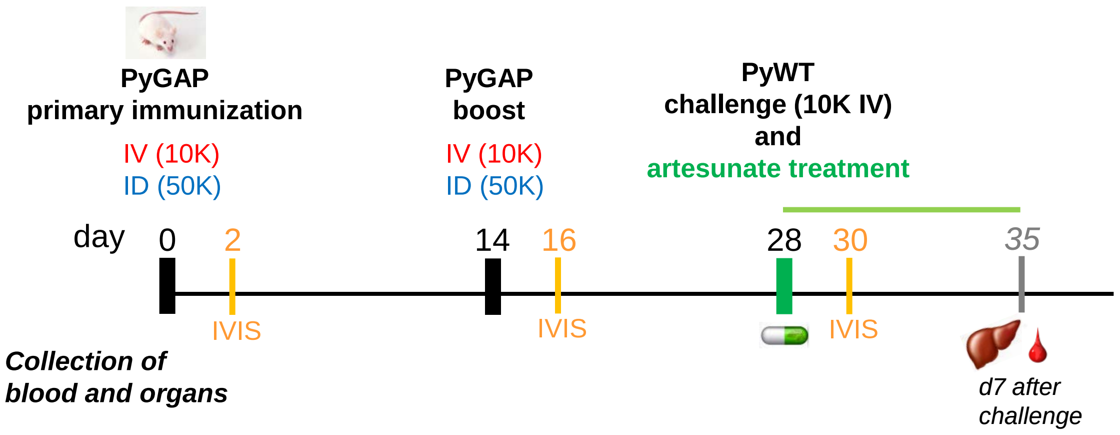

Figure S5. Comparable hepatic immune responses with and without artesunate (art) treatment

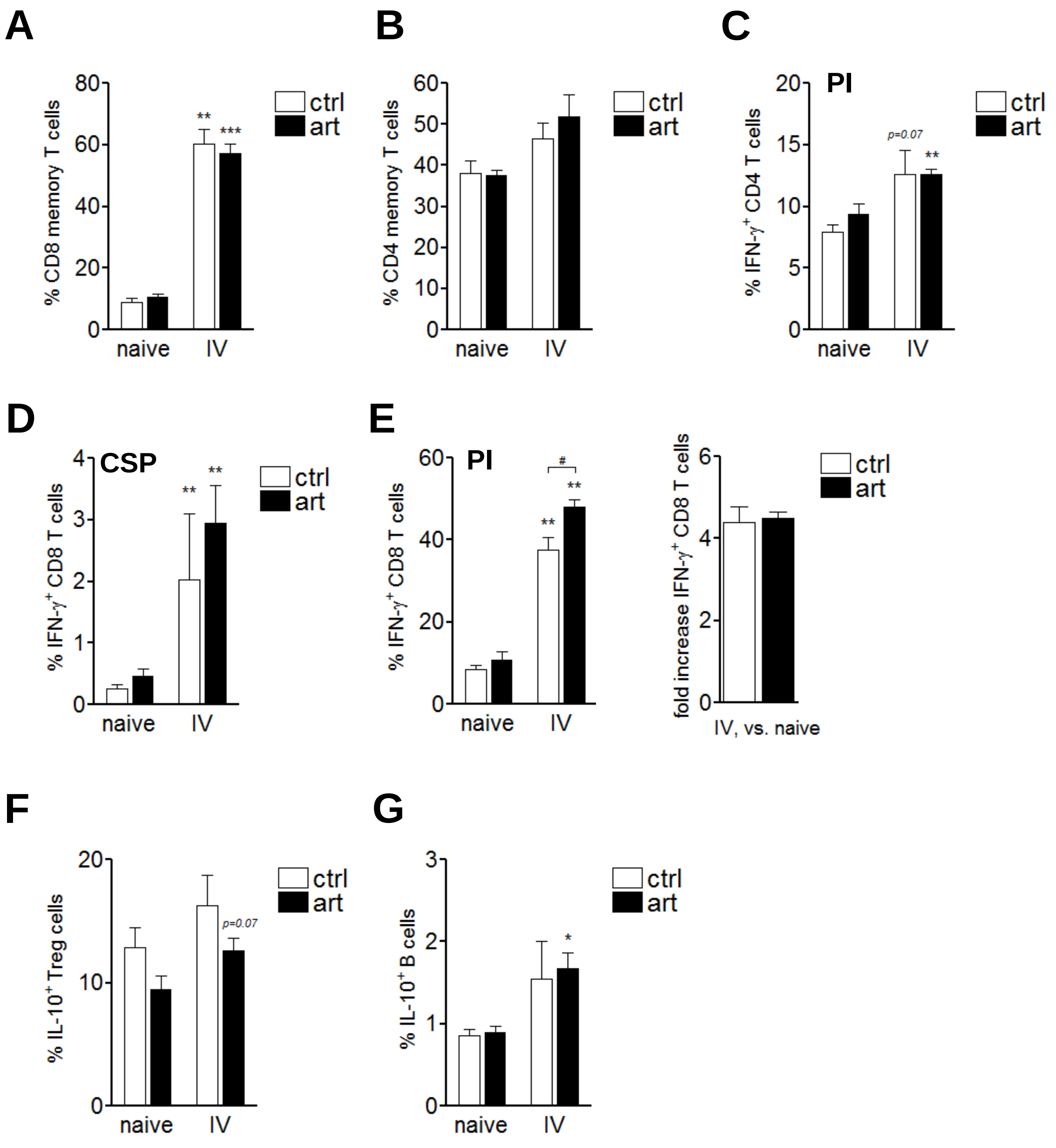

Figure S6. Effector responses that were mostly similar between protected and unprotected ID immunized mice

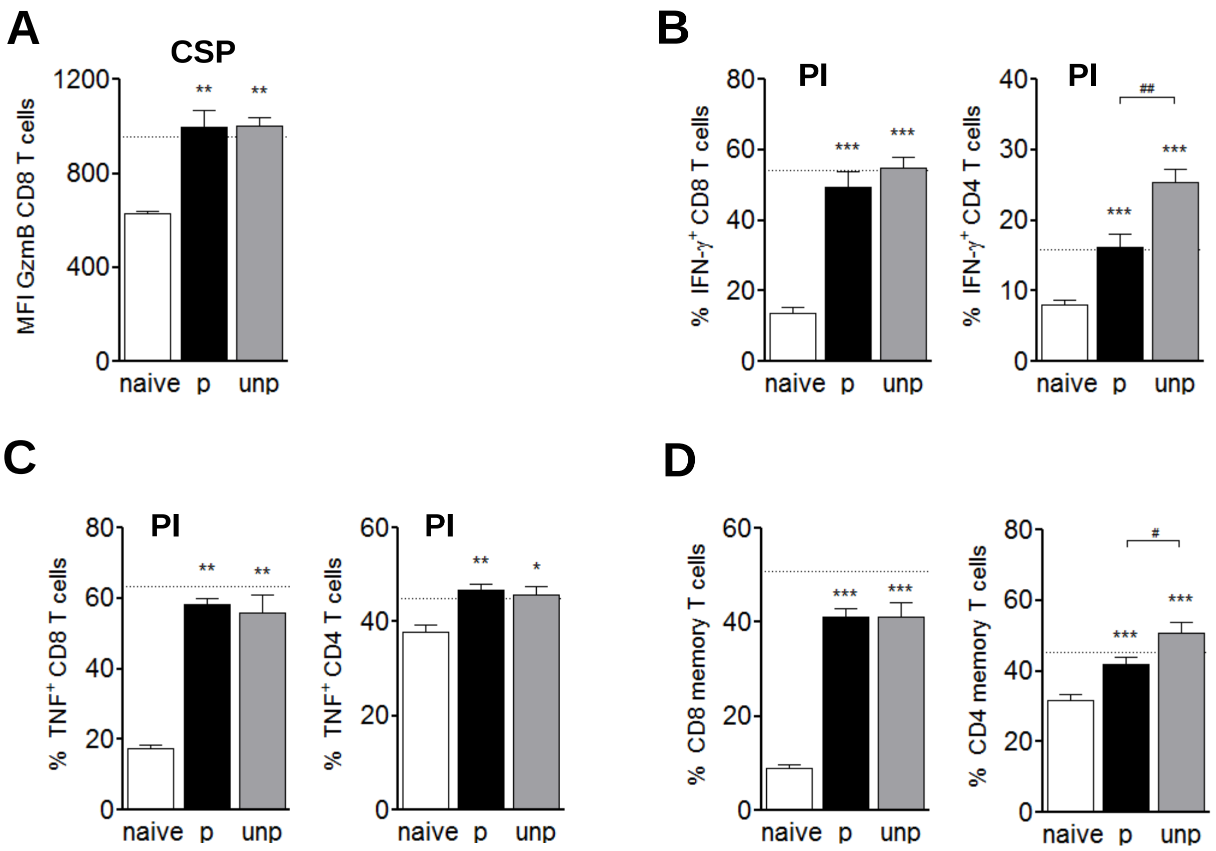

Figure S7: Effector immune responses in PBMC in protected and unprotected ID immunized mice

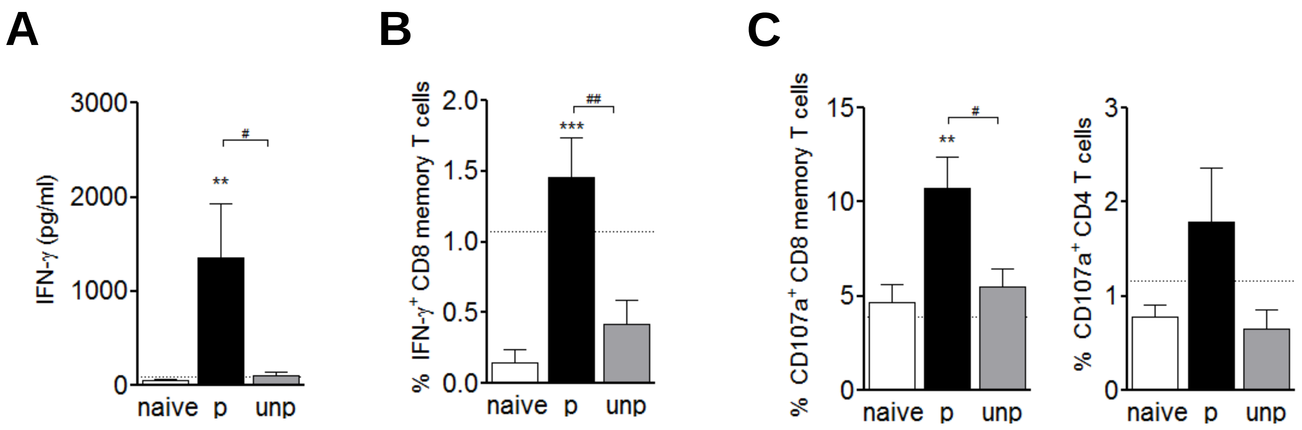

Figure S8. CSP-antibody titers in sera of naïve and IV and ID immunized mice

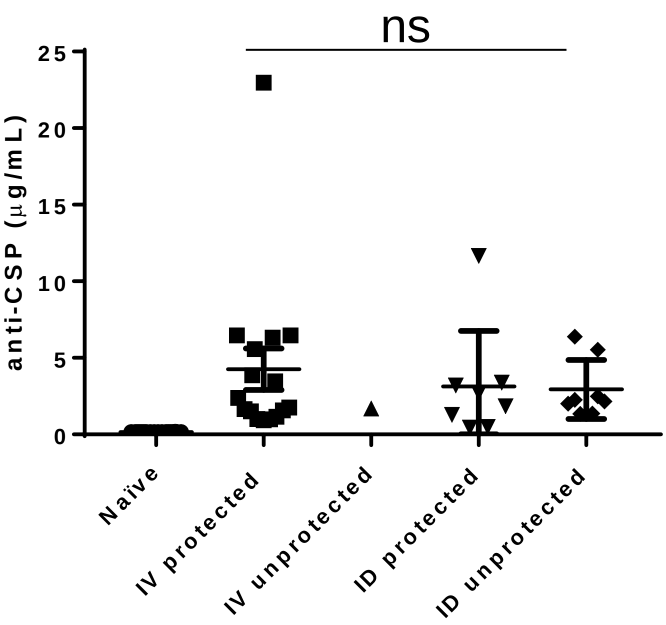

Figure S9: Regulatory responses which were similar between protected and unprotected ID immunized mice

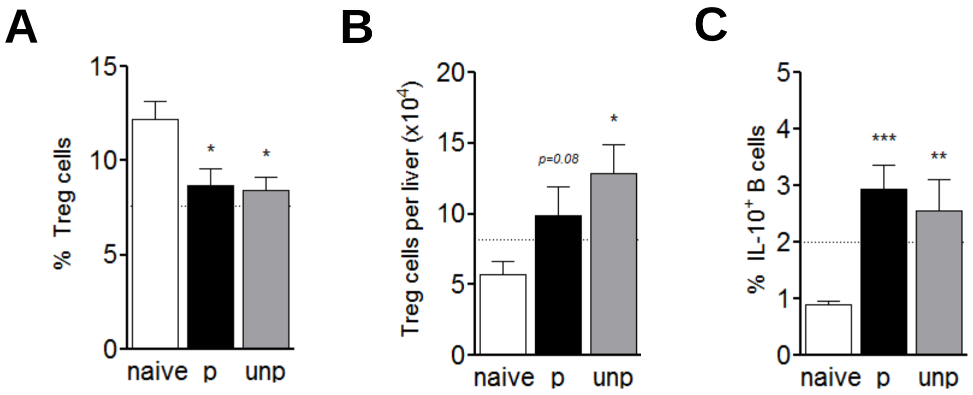

Figure S10: IV and ID route of immunization induce similar high T cell effector responses in liver

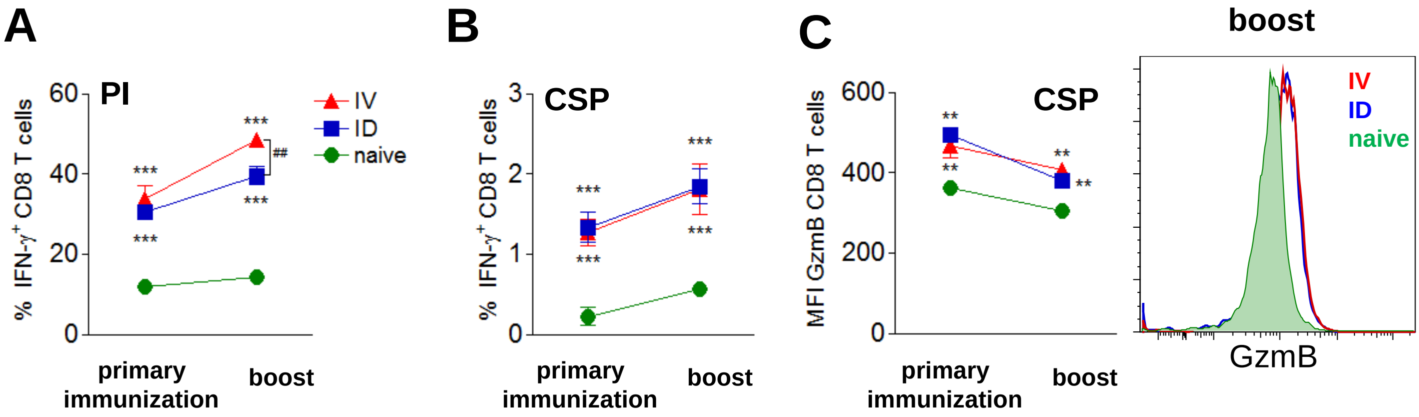

Figure S11: Exhaustion of hepatic T cells is comparable during IV and ID immunization

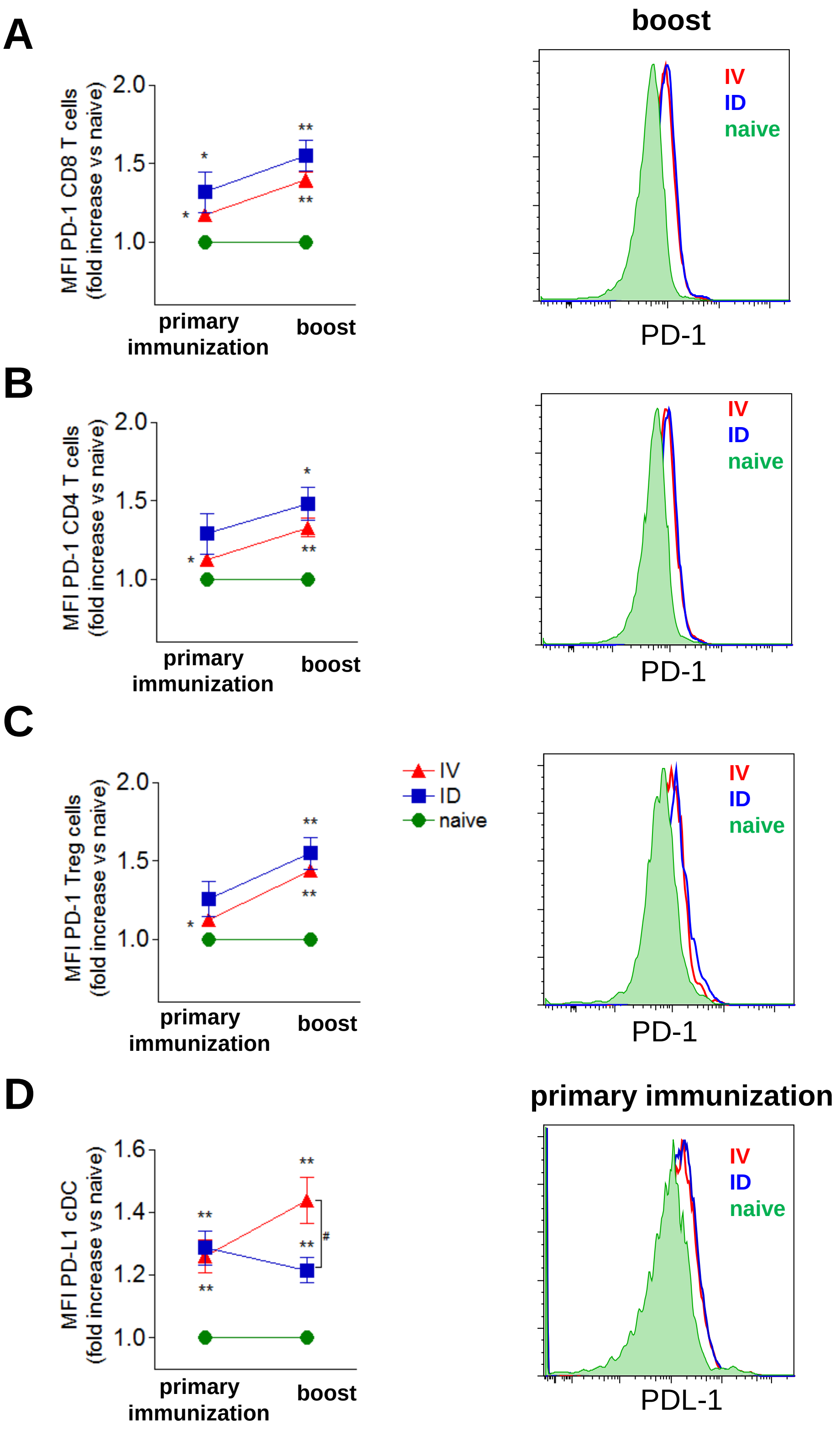

Figure S12: Regulatory marker expression on Foxp3-negative CD4 T cells after immunization

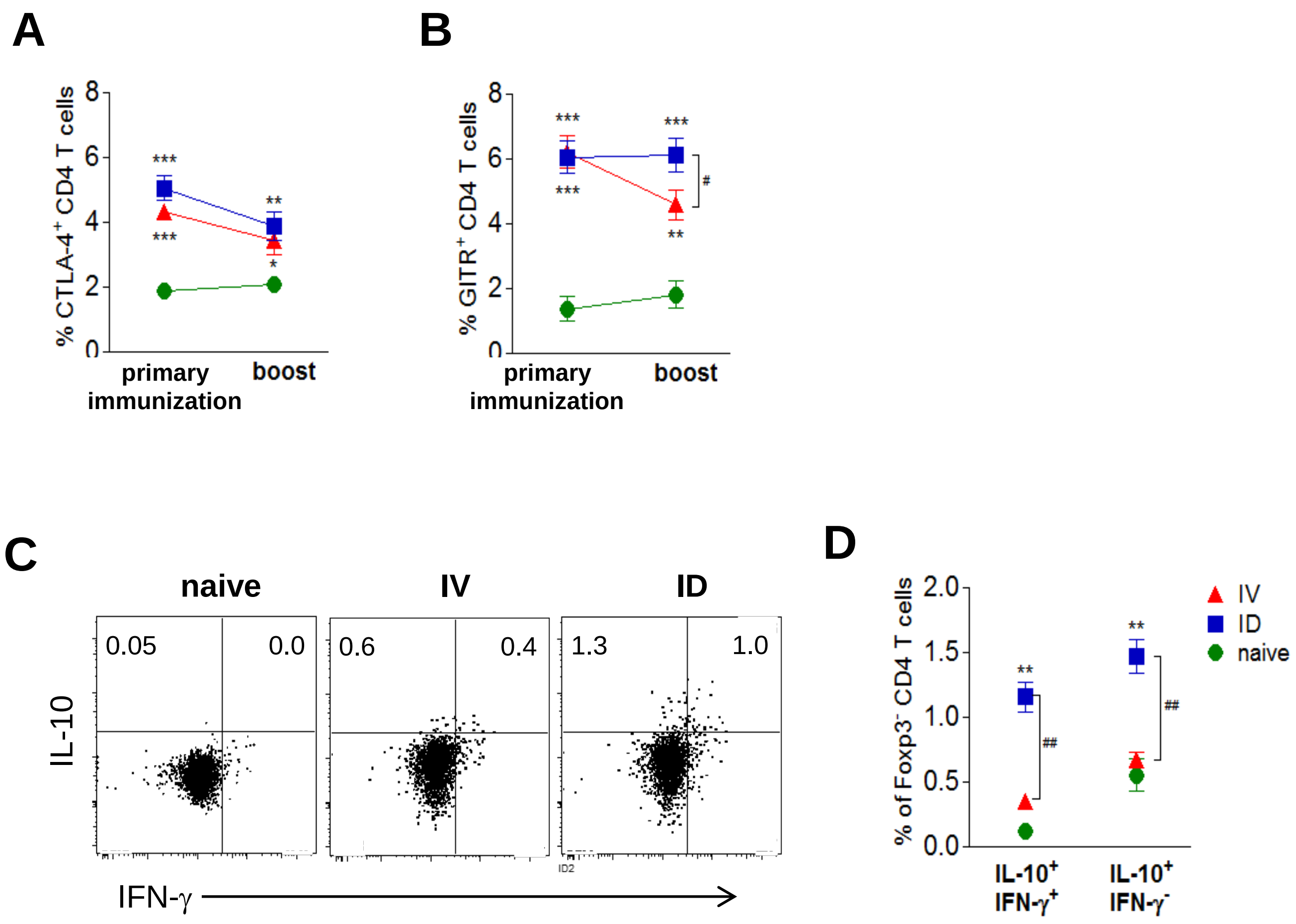

Supplementary table 1

Cell number of different cell types per liver at day 7 after primary immunization or boost by IV or ID route of sporozoite administration. Average of 5 mice per group ± SEM. *p*-value by Mann-Whitney is indicated by * $p<0.05$, ** $p<0.01$ between IV and ID.

**Population**		**Cell number day 7 after immunization**				**Cell number day 7 after boost**			
		**naive**	**IV**	**ID**	***p***	**naive**	**IV**	**ID**	***p***
Total liver leukocytes	$X10^6$	5.5±0.2	10.9±0.3	11.2±0.4		4.0±0.1	8.4±0.3	7.9±0.2	
CD44+ CD8 T cells	$X10^3$	7.2±0.2	46.9±1.7	52.3±2.8		3.6±0.2	68.3±3.5	38.9±2.1	*
CSP-specific IFN-g+ CD8 T cells	$X10^3$	0.0±0.0	2.1±0.2	2.9±0.4		0.3±0.01	2.7±0.3	2.9±0.3	
CTLA-4+ Foxp3- CD4 T cells	$X10^3$	4.0±0.4	28.1±0.8	46.7±2.7	**	2.1±0.2	14.3±1.0	14.8±0.9	
GITR-1+ Foxp3- CD4 T cells	$X10^3$	1.9±0.3	37.6±1.3	46.9±1.7		1.0±0.1	15.8±1.3	14.2±1.0	
IL-10+ Foxp3- CD4 T cells	$X10^3$	1.5±0.1	3.9±0.1	13.4±0.7	**	0.2±0.0	0.9±0.1	1.0±0.1	
IL-10+ B cells	$X10^3$	12.9±0.4	13.2±0.9	20.4±1.8		8.0±0.4	11.4±0.7	12.1±1.0	

Supplementary table 2

Frequencies of different cell types in lymph nodes at day 7 after primary immunization via IV or ID route of sporozoite administration. Average of 10 mice per group ± SEM. *p*-value by Mann-Whitney is indicated by * *p*<0.05, ** *p*<0.01, *** p<0.001 between IV and ID.

**Population**	**Frequency day 7 after immunization**			
	**naive**	**IV**	**ID**	***p***
Total CD19+ B cells	20.6±1.2	17.0±2.3	27.6±3.0	*
Total CD4 T cells in CD3+ T cells	76.9±5.3	76.0±5.3	65.3±9.1	ns
Total CD8 T cells in CD3+ T cells	9.8±1.2	8.1±1.1	8.7±0.9	ns
IFNg+ of CD4 T cells	0.6±0.1	0.5±0.0	3.5±0.6	***
IFNg+ of CD8 T cells	1.4±0.1	1.4±0.1	19.0±2.7	***
IL10+ of CD19+ B cells	0.6±0.1	0.6±0.1	1.8±0.2	***
IL10+ of Foxp3- CD4 T cells	0.4±0.1	0.3±0.0	1.1±0.3	***
IL10+ of Foxp3+CD25+ Tregs	1.5±0.3	1.3±0.2	4.8±1.0	**
CTLA-4+ of Foxp3- CD4 Tcells	0.5±0.1	0.4±0.0	1.4±0.2	***
CTLA-4+ of Foxp3+CD25+ Tregs	34.5±0.9	33.5±0.9	40±1.2	***
